# Bayesian optimisation of restriction zones for bluetongue control

**DOI:** 10.1038/s41598-020-71856-4

**Published:** 2020-09-15

**Authors:** Thomas Spooner, Anne E. Jones, John Fearnley, Rahul Savani, Joanne Turner, Matthew Baylis

**Affiliations:** 1 Department of Computer Science, University of Liverpool, Liverpool, UK; 2 Department of Mathematical Sciences, University of Liverpool, Liverpool, UK; 3 Department of Epidemiology and Population Health, University of Liverpool, Liverpool, UK; 4 IBM Research, The Hartree Centre, STFC Daresbury Laboratory, Sci-Tech Daresbury, Warrington, WA4 4AD UK

**Keywords:** Epidemiology, Epidemiology, Computational science, Computational science, Computer science

## Abstract

We investigate the restriction of animal movements as a method to control the spread of bluetongue, an infectious disease of livestock that is becoming increasingly prevalent due to the onset of climate change. We derive control policies for the UK that minimise the number of infected farms during an outbreak using Bayesian optimisation and a simulation-based model of BT. Two cases are presented: first, where the region of introduction is randomly selected from England and Wales to find a generalised strategy. This “national” model is shown to be just as effective at subduing the spread of bluetongue as the current strategy of the UK government. Our proposed controls are simpler to implement, affect fewer farms in the process and, in so doing, minimise the potential economic implications. Second, we consider policies that are tailored to the specific region in which the first infection was detected. Seven different regions in the UK were explored and improvements in efficiency from the use of specialised policies presented. As a consequence of the increasing temperatures associated with climate change, efficient control measures for vector-borne diseases such as this are expected to become increasingly important. Our work demonstrates the potential value of using Bayesian optimisation in developing cost-effective disease management strategies.

## Introduction

Bluetongue (BT) is a viral disease of ruminants (including cattle and sheep) that is transmitted by species of Culicoides biting midges. BT has dramatically expanded its geographical range in recent decades, spreading across southern Europe from 1998 and occurring for the first time in northern Europe in 2006^1^. BT is climate-dependent due to its exothermic midge vector and the recent expansion has been linked to climate change^2,3^. Previous outbreaks of BT in Europe have had a severe impact on the farming industry. In the Netherlands alone, outbreaks have cost the economy hundreds of millions of USD through animal mortality and morbidity, the costs of control and, in particular, lost trade^4^. BT is considered an ongoing threat in the UK and Europe^5,6^, with effective control measures anticipated as being increasingly vital to prevent devastating outbreaks^7^. In 2015, BT reappeared in France and overwintered, with two strains of the virus, BTV-8 and BTV-4, currently circulating^8,9^.

The first and to date only outbreak of BT in the UK occurred in 2007. The introduction of the virus is generally considered to have been caused by wind-borne midges moving from mainland Europe to south-east England on the night of the 4th/5th August^10^. This outbreak was constrained to south-east England, with only 135 farms being affected, a small number compared with mainland Europe ($\sim 40{,}000$ affected farms). The small scale of the spread has been attributed to three primary factors^11^: (1) the area of introduction had a relatively low density of farms and thus fewer opportunities for between-farm vector transmission; (2) recorded temperatures compared with previous years and that experienced in Europe were moderate; (3) the pre-existence of animal movement restrictions due to foot-and-mouth disease. These circumstances proved to be fortunate for the UK, but this is unlikely to be the case in the future. Indeed, BTV-8 has since been detected in UK farms through routine post-import testing of cattle from France^12^ and, while no onward spread has been observed, BT represents an active threat to farms in the UK.

Transmission of BT occurs via two routes: the dispersal of infectious midges (either via free flight or wind-borne) and the movement of infected animals between farms (either directly or via markets). Given the potential for long range transmission to uninfected areas by trade networks, the movement of animals is tightly controlled. All EU member states are required to enforce restrictions on animal movements once a BT outbreak has been confirmed. See, in particular, Commission Regulation (EC) No 1266/2007 of 26 October 2007 on implementing rules for Council Directive 2000/75/EC with regards to the control, monitoring, surveillance and restrictions on movements of certain animals of susceptible species in relation to bluetongue. In the UK, these restrictions are enforced around detected farms according to policy dictated by the Department for Environment, Food and Rural Affairs (Defra)^13^: once an infection is detected, there is a complete ban on movement for all farms within a radius of 20 km of the affected farm. This area is known as the **Control Zone** (CZ). Outside this, two zones are defined that correspond to two decreasing levels of infection risk: a **Protection Zone** (PZ), which extends to a radius of 100 km beyond the detected farms, and a **Surveillance Zone** (SZ) which occupies a further 50 km outside the PZ. Within PZ and SZ, animal movements are allowed only towards a zone of equal or higher risk, and only when the movement does not involve crossing a disease-free area. The sizes of these three regions will be the primary focus in this work. They will generally be referred to by the *control radii*: $r_\text {CZ}$, $r_\text {PZ}$ and $r_\text {SZ}$, respectively, where $r_\text {CZ}< r_\text {PZ}< r_\text {SZ}$ by definition.

### Related work

Previous modelling studies on BT have primarily focused on the impact of control via vaccination^14–16^. Investigations into optimal movement restrictions, and the impact of policy on the control of bluetongue, has been somewhat limited in comparison. For example, Sumner et al.^17^ used a simulation model to study the efficacy of animal movement restrictions in controlling BT and the Schmallenberg virus (SBV, also spread by Culicoides biting midges). They found optimal movement restriction zone sizes for BT in the range 20–40 km depending on the distribution of vector flight distances. Most recently, work by Jones et al.^7^ and Turner et al.^11^ built on the model of Turner et al.^18^ to explore the factors contributing to the 2007 UK bluetongue outbreak and the potential increase in risk associated with changes in climate. In both cases, movement restrictions were found to be an effective method for controlling the spread of infection.

Similar studies have also been conducted for animal diseases besides bluetongue. For example, British policy changes were studied by Vernon and Keeling^19^, who considered the epidemic potential of the cattle movement network in Britain between 2000 and 2009 as more stringent regulations on cattle movements were introduced. Their study observed a decrease in risk associated with the majority of policy changes. Thulke et al.^20^ also found that movement restrictions were more impactful in their model than pre-emptive culling in limiting the spread of classical swine fever in pigs. Mohr et al.^21^ recently looked at the impact of altering the contact network structure of livestock in Scotland on simulated foot-and-mouth disease dynamics. Optimal control measures have been studied extensively for plant diseases as well, even more so than for animal diseases. Typically optimisation has been performed over a single control parameter, e.g. treatment or culling radius over a range of values. For example, Cunniffe et al.^22^ optimised the treatment radius for sudden oak death in California as simulated by a stochastic epidemic model and, similarly, Cunniffe et al.^23^ optimised the cull radius for citrus canker in Florida.

Most recently, Bussell et al.^24^ discussed two techniques for bridging the gap between epidemic simulation and optimal control through the use of optimisation. In a similar vein of work, Probert et al.^25^ addressed the need for context in outbreak response strategies using reinforcement learning. Morgan^26^ suggest that methods such as these are already a crucial tool to be incorporated into decision-making on epidemiological control. In this paper we complement these results and present a Bayesian framework for the control of vector-borne diseases.

### Our contributions

In this paper we tackle the problem of bluetongue control using an optimisation framework for deriving cost-effective policies without direct access to the underlying model of BT. We use an existing stochastic simulator for bluetongue outbreaks^7,11,18^ and leverage access to animal movement and climate data to evaluate our approach with a foundation in real, historical events. We begin with an evaluation of two variants of the UK government’s current bluetongue control policy^13^. We show how the current use of a total movement ban in the control zone reduces the number of farms and the maximum distance of the spread in expectation. This is explored for outbreaks starting anywhere in England and Wales, and in specific regional cases. Next, we introduce an optimisation framework for deriving radii that minimise the expected number of infected farms while using only two zones instead of three, and which allow movement of animals in the control zone; i.e. a much simpler policy. It is shown that these are as effective as the government’s current approach despite requiring fewer restrictions on the movement of animals. We present evidence that these results are consistent across both national and regional levels, and that they generalise to unseen climate and animal movement data. Finally, we introduce a pseudo-economic cost model that takes into account the impact of each newly infected farm as well as the perpetual cost of enforcing movement restrictions. The potential economic impact of each policy is then explored along with a sensitivity analysis of the proposed method to perturbations in the control radii, introduction date of the outbreak, and spatial features of each region. Our results show that Bayesian optimisation is well suited to solving epidemiological problems and may be easily integrated into existing frameworks.

## Methods

Our approach makes use of machine learning and Bayesian optimisation to find control policies that are simpler and cheaper to implement. The proposed method follows a typical optimisation loop: (1) query a set of parameters that satisfy the problem constraints; (2) run simulations to evaluate the performance measure for the chosen parameters; (3) use this information to update a surrogate model of the expected output, and repeat until a tolerance on performance or computational resources is reached; (4) find the best performing point in our observation set and return. This type of optimisation is especially well suited to problems which take a long time to sample, those with continuous domains, and those with random variation between evaluations. By maintaining a surrogate model over the objective, Bayesian optimisation is able to quantify its own uncertainty and thus reason about where to try next in a highly efficient manner. The key components needed by this process are summarised in the following sections; see “Optimisation model” section  for further technical details.

### Bluetongue simulations

BT outbreaks were simulated in England and Wales using a stochastic-dynamic network model of BT transmission between farms. The simulator was developed initially for eastern England^18^ but was recently improved and extended to cover all of England and Wales^11^. In the model, individual farms are represented as being in one of the following disease states: susceptible, exposed, infectious or detected. Disease transmission between farms is modelled as a stochastic process via two routes: (1) continuous diffusion of infectious vectors from infectious farms, and (2) movement of infectious or exposed animals using a daily time-series of observed animal movement data from a recent disease-free year (2013). At the farm level, disease prevalence of infected farms is modelled deterministically and used to define that farm’s infectivity on any given day following infection. Both inter- and intra-farm model components incorporate temperature-dependent parameters (see supplementary material for more details), each of which is calculated from the 5-day moving average of daily temperatures at the farm location. Defra standard movement restrictions were employed in the default model set up. The model has previously been validated by comparing the spatial and temporal patterns of outbreaks simulated for 2007 with the location and detection dates of farms which were affected in the 2007 outbreak^11^.

Each sample outbreak was generated by the simulator using the following process:

1. A simulation is initiated on day 1 (1 January) and progresses one day at a time until day 365, when it ends.
2. On day $T_\text {intro} = 121$ (1 May) of the simulation, an outbreak is initiated with the infection of a single animal (a cow for cattle farms and either a cow or sheep with equal probability on mixed farms) in a randomly-chosen farm in the target region. This day was chosen specifically to ensure the size of the outbreak was large^18^ and provide a more substantial scenario upon which to evaluate our proposed method.
3. The spread of infection between farms due to vector dispersal and animal movements is simulated at each time step. The dynamics of this process, including the probability of a farm being detected, are governed by the model parameters listed in the supplementary material.
4. Upon detection, fixed-rule control zones are enforced around each and every quarantined farm, within which animal movement is restricted. The radii of these zones are dictated by the choice of policy and remain fixed for the remainder of the simulation (i.e. remainder of the year).

With repeat sampling (each using different random seeds), one can obtain a distribution over various metrics of interest—such as the number of infected farms and peak spread distance—as a function of the chosen simulator parameters; this process is commonly referred to as Monte Carlo (MC) sampling and can be used to construct unbiased estimators. In this work, we focus on three key variables of the simulator that impact behaviour:

**Control policy**: The rules dictating how the three restriction zones are enforced.
**Starting region**: The region of the UK in which the first infection occurs; taken as either a random region or explicitly chosen from one of the 7 regions in Table 1.
**Animal movements**: A dataset of animal movements between farms; typically based on a single year of real data.
**Climate conditions**: A dataset of temperature readings from across the country; again, typically based on a year of real data.

Simulations using movement data from 2006/2013, and temperature data from 2006/2013–14, were used to form the main body of our results. In general, we will refer to the configuration used when evaluating a policy as the *test conditions*, and those used during optimisation (i.e. to learn a policy) as the *train conditions*. This distinction is crucial, since one may only draw conclusions about performance on data unseen during training. The specific metrics used to perform this analysis will be defined in “Performance metrics” section.

### Control policies

Three different policies for restricting animal movements were considered. The first is a replica of the UK government’s current policy as outlined by Defra^13^. The second is a variation that allows movement within the control zone but otherwise maintains three zones and the same radii as in the first policy; while a seemingly subtle change, this will turn out to be very impactful on performance. The third policy relaxes our constraints even further by having only two zones and control radii derived by optimisation. These three policies will be referred to as the following:

**NMIZ**: **N**o **M**ovement in the **I**nnermost **Z**one—this is the containment policy specified by government documentation as referred to previously: a *3-zone policy with hard constraints in the innermost zone* and radii of size
  1 $$\begin{aligned} r_\text {CZ}= 20\,\text {km},\ r_\text {PZ}= 100\,\text {km}\ \text {and}\ r_\text {SZ}= 150\,\text {km}, \end{aligned}$$
  for the control, protection and surveillance zones, respectively.
**MIZ**: **M**ovement in the **I**nnermost **Z**one—this is a variant of NMIZ in which *movement of animals is allowed in the control zone*. The same radii of 20 km, 100 km and 150 km for the control, protection and surveillance zones, respectively, are used.
**OPT**: **Opt**imisation-based—these policies use only *two zones* and, as with MIZ, *allow movement within the innermost (control) zone*. They are derived by minimising the expected number of infected farms when sampling the simulator with respect to the choice of radii. We will also refer to these as the $J^\text {NI}$-optimised policies, where $J^\text {NI}$ is defined as the number of infected farms at the end of the year.

Following the GB Bluetongue control strategy, these zones are placed around all farms that have, at any point in time, been infected and detected. “The Restricted Zone will [then] remain in place and measures will continue to be implemented until amended or repealed by the relevant administration with the approval of the Commission”^13^; note that the latter condition is not something that can be built into a simulator. While each outbreak is treated on a case-by-case basis, it is reasonable to assume that the zones would stay in place until the spread of infection has subsided.

### Performance metrics

Three metrics were used to evaluate a policy: (1) the number of unique farms infected during the simulation, (2) the maximum distance from the locus of the spread to a case of infection in kilometres, and (3) the cumulative pseudo-economic cost of the policy. We denote the value of these objectives at time *t* by $J^\text {NI}_t$, $J^\text {MS}_t$ and $J^\text {EC}_t$, respectively. To define $J^\text {EC}_t$, we denote the total number of farms in any movement constrained zone at a time *t* by $Z_t$. We then define zone-specific farm counts by the addition of a superscript that distinguishes the zone. For example, $Z_t^\text {CZ}$ refers to the number of farms in the *control zone* at time *t*. The number of infected farms at time *t* is denoted by $N_t$, and the change since the previous time $t-1$ as $\Delta N_t \equiv N_t - N_{t-1}$. The cost incurred at each time step may then be defined for $t > 0$ by the following weighted summation,

2 $$\begin{aligned} X_t = \underbrace{w_\Delta \cdot \max \left[ \Delta N_t, 0\right] }_\text {Immediate term} + \underbrace{w_\text {CZ}\cdot Z_t^\text {CZ} + w_\text {PZ}\cdot Z_t^\text {PZ} + w_\text {SZ}\cdot Z_t^\text {SZ}}_\text {Running term}, \end{aligned}$$

which captures the immediate cost due to infection and the recurring costs due to containment. We refer to this as the *differential pseudo-economic cost*. Given $X_t$, the *cumulative pseudo-economic cost* may be defined simply by the linear difference equation below:

3 $$\begin{aligned} J^\text {EC}_t \equiv {\left\{ \begin{array}{ll} J^\text {EC}_{t-1} + X_t, &{} \text {for } t > 0, \\ 0, &{} \text {for } t = 0. \end{array}\right. } \end{aligned}$$

For brevity, we refer to the total cost incurred over the course of a simulation (i.e. by day 365) with the shorthand $J^\text {EC}\equiv J^\text {EC}_{365}$. This is equal to the summation over the differential costs accrued at each increment, starting from time $t=1$,

$$\begin{aligned} J^\text {EC}= \sum _{t=1}^{365} X_t. \end{aligned}$$

As noted in previous work^18^, the distributions of these metrics exhibit highly non-normal features including an excess of zeros, overdispersion, extreme values and skew. This is highly common for distributions of variables found in health, financial and geospatial settings. In epidemiology this often relates to the notion that an epidemic only occurs if a “threshold” is surpassed during the incubation period, escaping the initial basin of attraction towards dissipation. In order to prevent misleading results that may arise from poor point estimates, we make two assumptions. First, inspired by existing work in time series modelling for epidemiological phenomena^27,28^, each of the three objectives are assumed to be observations derived from some underlying process that is well described by a hurdle model^29^. This means that the sufficient statistics for a set of samples may be restricted to the distribution of non-zero values and the probability of no measurable spread, $\pi$. Since it was found in all cases that $\pi$ was independent of the choice of control radii, comparisons between policies were drawn using only the remaining data cases where the “hurdle” was overcome. The second simplification is to assume that the distributions of the non-zero values are unimodal. This allows us to use the median as a principled measure of central tendency and the interquartile range as an estimate of dispersion. These are more robust to extreme values than the mean and standard deviation, respectively, and allow for more accurate comparisons. The 95% confidence interval on each summary statistic, and any combinations of them, is estimated through bootstrap resampling, unless otherwise stated^30^.

#### Interpretation of $J^\text {EC}$

The *pseudo*-economic cost model used in this paper is intended to provide a relative measure by which to compare the effectiveness of a set of policies. We make no claim that it is sufficiently expressive to capture the economies at play in the agriculture industry; this, in and of itself, is a highly complex and nuanced problem. However, it is crucial when developing control methods that we are able to make “reasonable” comparisons. While the absolute values themselves cannot be compared to anything observed in the real world, they do facilitate critical analysis of our results. As is often quoted: “all models are wrong, but some are useful”^31^. Below we outline some of the underlying assumptions and areas for future work to build upon:

1. We assume that the weights are fixed and do not change with the size of the zone nor over time. While the impact of the latter is hard to quantify, relieving the former would impact strategies with especially large zones the most.
2. We also assume that the cost incurred to a farm in a given zone is the same, independent of the size or nature of the farm in question. This is certainly an important consideration, though calibrating such a model is beyond the scope of this work.
3. We do not account for seasonalities in market demand. This may have the effect of inflating “cost” in periods of low trading activity and under-representing it when activity would have otherwise been at its peak.

## Results

**Figure 1:**
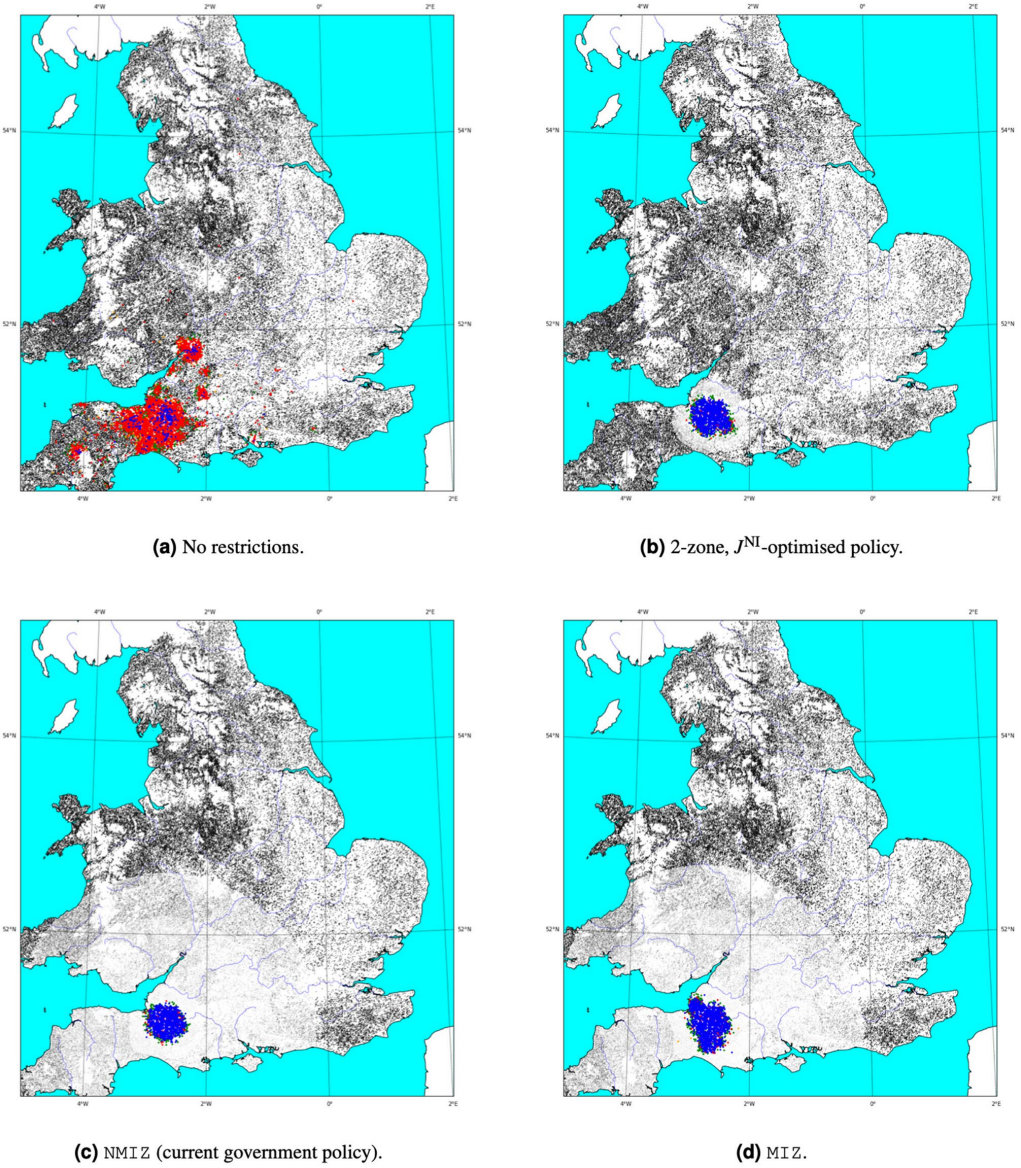
Maps showing the dispersion of infections across England and Wales at the peak of each spread (27 October—day 300) during a simulation starting with an infection of a single animal on May 1st. For each policy, the simulation was initiated with the infection of the same farm in Somerset and the same set of parameters governing disease propagation. Each point represents a single farm where the colour indicates the following states: black—susceptible; green—exposed; red—infected; blue–infected and detected. Susceptible farms within restriction zones are shaded with increasingly lighter greys as the occupying area transitions from outer to inner zones; or equivalently, from lower to higher risk.

We begin by exploring the potential impact of a bluetongue outbreak when no preventative measures are in place. An example of such a scenario is illustrated in Fig. 1. This shows how the absence of movement restrictions can lead to: (a) multiple dense clusters of infected farms in south-west England, as well as; (b) a scattering of affected farms covering the majority of England, including much of Cornwall, and even as far north as Leeds. These observations reflect the two modes of transmission handled by the model, that of farm-to-farm movement and vector dispersal, respectively. While the latter is a local phenomenon, at least on land, with the majority of cases occurring within 31 km of the host^32^, the former can occur over long distances and is often responsible for the emergence of new infection loci. This example corroborates past work^7,18^ and suggests that the potential impact of a bluetongue outbreak could be widespread and severe.

The results presented hereafter focus on containment policies designed to subdue the propagation of an outbreak following first detection. All three control policies—NMIZ, MIZ and OPT—will be evaluated on both national and regional levels (see Table 1). Each such *outbreak begins* with the infection of a single animal in *a randomly selected* region of England or Wales (national), versus only a *single region* in each simulation (regional). The former addresses the need for a policy that is effective at preventing the onward spread of BT, independent of where the infection begins. The latter addresses efficiency—i.e. can more effective policies be found given knowledge of the *region of origin*; this makes the reasonable assumption that animal movements in the early phases of the spread have a significant impact on how it develops in the long run.

**Table 1:** Spatial features derived from shape files of the 7 regions (counties) considered in this paper.

Region	Area (km$^2$)	Farms		Animals	
		Count (–)	Density (km$^{-2}$)	Count ($10^5$)	Density (km$^{-2}$)
Cheshire	2,338	2,820	1.21	4.48	192
East Sussex	1,784	1,602	0.90	3.06	172
Hampshire	3,741	1,886	0.50	2.02	54
Norfolk	5,334	1,864	0.35	2.12	40
Somerset	4,176	5,391	1.29	9.30	223
Dyfed	5,702	8,255	1.45	22.7	398
Cumbria	6,777	5,373	0.79	25.3	373
England	1.30 $\times$ 10^5^	9.20 $\times$ 10^4^	0.71	216	166
Wales	2.06 $\times$ 10^4^	2.29 $\times$ 10^4^	1.11	95.1	476

### Benchmark policies

#### National

Our first benchmark was generated by evaluating the NMIZ and MIZ policies on outbreaks starting in any region in England or Wales. Data on the movement of animals and recorded temperatures collected in 2013 were used to generate 1,000 random outbreaks from the simulator. The performance of each policy is summarised by the central tendency using point estimates for the median value observed for each metric, ignoring cases where the outbreak failed to propagate. Confidence intervals on these values were then estimated using bootstrapping^30^. The median number of infected farms $J^\text {NI}$, maximum lateral spread distance (which we denote by $J^\text {MS}$) and economic cost (for which the chosen weights are defined in “Economic impact” section) are quoted in Table 2.

**Table 2:** Central tendency for the number of infected farms, maximum spread distance and economic cost for outbreaks originating in regions in England and Wales using movement and temperature data from 2006 and 2013.

Policy	Train year	Test year	Infected farms (–)	Spread distance (km)	Economic cost ($10^6$)
NMIZ	–	2006	$1604_{-186}^{+161}$	$35.78_{-0.71}^{+1.27}$	$10.55_{-0.49}^{+0.62}$
		2013	$714_{-109}^{+106}$	$29.23_{-1.51}^{+2.49}$	$8.96_{-0.60}^{+0.46}$
MIZ	–	2006	$1721_{-214}^{+189}$	$50.21_{-2.11}^{+3.19}$	$11.06_{-0.50}^{+0.91}$
		2013	$744_{-110}^{+113}$	$39.79_{-3.77}^{+2.27}$	$9.27_{-0.45}^{+0.78}$
OPT	2006	2006	$1598_{-188}^{+175}$	$35.74_{-1.02}^{+1.20}$	$1.34_{-0.15}^{+0.07}$
		2013	$717_{-93}^{+116}$	$30.08_{-1.52}^{+1.59}$	$0.81_{-0.09}^{+0.09}$
	2013	2006	$1630_{-215}^{+176}$	$37.09_{-1.73}^{+1.01}$	$0.80_{-0.09}^{+0.06}$
		2013	$723_{-107}^{+97}$	$29.73_{-1.79}^{+2.16}$	$0.52_{-0.06}^{+0.05}$

Each row corresponds to a different policy on the restriction of animal movements. Estimates are given by the median of 1,000 samples and are quoted with the 95% confidence interval derived from bootstrapping.

We found that the NMIZ policy produces the best results in terms of minimising the spread, but does so at great economic cost. Using the same radii but relaxing movement restrictions in the innermost zone led to a significant increase in both metrics. This is particularly true of the spread distance. It is apparent that the dynamics of BT in zones where movement is allowed is very different from those in which farms are placed under a total movement ban. This observation was reflected in all three metrics: the number of infected farms, spread distance and cost. Interestingly, the MIZ policy has high expected economic cost as well, despite the fact that the relaxed constraints on the control zone incur lower per-farm expenses. This hints at the existence of “optimum” policies for which there is a trade off between performance and cost effectiveness. In other words, allowing more movement of animals within the three zones alone—and in so doing, negatively affecting performance—is not sufficient to improve economic efficiency. Instead one must find policies that not only relax constraints, but also match the performance of NMIZ. Otherwise, the increased size of outbreaks leads to the very same large costs we are trying to avoid.

#### Regional

We next evaluated the NMIZ and MIZ policies on outbreaks starting in the 7 regions in Table 1 to see more precisely how the discrepancy between the two approaches depends on the region of introduction. Again, movement and temperature data from 2013 were used to generate sample outbreaks; this time using 250 samples per region. Outbreaks were also simulated using movement data from 2013 with temperature data from 2014 to explore the significance of climate; this has been addressed in more detail by Jones et al.^7^. As in the national case, the difference in the number of infected farms between the two policies is less pronounced than that observed for the spread distance (see Tables 3 and 4). Most regions (excluding Somerset) show practically indistinguishable medians for the number of infected farms for the two policies—though NMIZ is marginally lower on average. This observation is reflected in previous work^18^ showing that targeted restrictions are often as effective as imposing a unilateral ban over movement for every farm. On the other hand, we see that NMIZ generates a much lower value for $J^\text {MS}$. In Dyfed, for example, the relaxation of restrictions in the control zone leads to an increase in the median of $\sim 50\%$. Comparing Fig. 1 makes this especially clear. NMIZ mitigates long-range dispersal and helps to localise the spread in much smaller areas than MIZ. It is perhaps unsurprising that the size of the affected areas under movement restrictions are typically dominated by the range of vector dispersal.

**Table 3:** Central tendency for the number of infected farms and maximum spread distance for 7 regions in the UK using movement data and temperature from 2013.

Region	Infected farms (–)		Spread distance (km)	
	MIZ	NMIZ	MIZ	NMIZ
Cheshire	$1192_{-188}^{+161}$	$1182_{-194}^{+97}$	$53.38_{-7.82}^{+16.38}$	$45.51_{-10.72}^{+22.41}$
East Sussex	$1146_{-45}^{+77}$	$1094_{-71}^{+83}$	$45.51_{-3.78}^{+3.34}$	$30.88_{-1.65}^{+1.35}$
Hampshire	$548_{-53}^{+68}$	$556_{-70}^{+47}$	$32.54_{-2.49}^{+1.64}$	$28.18_{-1.10}^{+1.46}$
Norfolk	$248_{-48}^{+46}$	$248_{-47}^{+53}$	$25.10_{-1.07}^{+3.03}$	$24.03_{-0.80}^{+1.83}$
Somerset	$2693_{-256}^{+199}$	$2500_{-258}^{+157}$	$60.95_{-4.99}^{+7.29}$	$46.34_{-9.23}^{+11.15}$
Dyfed	$1258_{-175}^{+251}$	$1203_{-173}^{+184}$	$57.75_{-6.95}^{+7.39}$	$37.31_{-6.67}^{+12.06}$
Cumbria	$317_{-90}^{+70}$	$312_{-73}^{+99}$	$39.21_{-4.87}^{+14.26}$	$26.85_{-5.71}^{+13.74}$

Two variants on the government policy are quoted: with (NMIZ) and without (MIZ) movement restrictions in the control zone. Estimates are given by the median of 250 samples and are quoted with the 95% confidence interval derived from bootstrapping.

**Table 4:** Central tendency for the number of infected farms and maximum spread distance for 7 regions in the UK using movement data from 2013 and temperature data from 2014.

Region	Infected farms (–)		Spread distance (km)	
	MIZ	NMIZ	MIZ	NMIZ
Cheshire	$1416_{-102}^{+106}$	$1399_{-98}^{+78}$	$52.06_{-4.57}^{+3.63}$	$33.75_{-2.77}^{+16.75}$
East Sussex	$1690_{-64}^{+47}$	$1577_{-43}^{+56}$	$50.17_{-1.72}^{+1.64}$	$35.86_{-1.30}^{+0.91}$
Hampshire	$768_{-65}^{+83}$	$762_{-78}^{+88}$	$38.84_{-1.55}^{+3.33}$	$32.63_{-1.35}^{+1.75}$
Norfolk	$365_{-54}^{+62}$	$364_{-50}^{+46}$	$33.74_{-2.73}^{+3.48}$	$28.20_{-1.29}^{+1.01}$
Somerset	$3253_{-302}^{+298}$	$2863_{-129}^{+200}$	$61.45_{-4.11}^{+3.81}$	$43.13_{-3.89}^{+9.24}$
Dyfed	$1540_{-263}^{+163}$	$1387_{-176}^{+225}$	$55.56_{-4.25}^{+7.03}$	$32.36_{-1.43}^{+4.72}$
Cumbria	$339_{-141}^{+147}$	$318_{-118}^{+157}$	$45.86_{-7.69}^{+8.33}$	$26.50_{-4.23}^{+17.44}$

Two variants on the government policy are quoted: with (NMIZ) and without (MIZ) movement restrictions in the control zone. Estimates are given by the median of 250 samples and are quoted with the 95% confidence interval derived from bootstrapping.

A natural question to ask of these results is why regions behave differently. Tables 3 and 4 show that there is indeed significant variation across regions and between 2013 and 2014 temperature datasets. Not only that, there is also a great deal of variation in the effective difference between NMIZ and MIZ—though it is interesting to note that this effect does not appear to be uniform across all regions. One might infer that this is due to the relationships between the geospatial features of a region and the processes governing the propagation of BT. An important consequence which we shall address (“Strategy optimisation” section) is whether this relates to the amenability of a region to our optimisation-based approach.

### Strategy optimisation

In this section we explore the performance of optimisation of the 2-zone policy OPT. Experimental evidence indicates that when movement is allowed in the control zone—as is the case here—then the choice of radii plays a more important role in our ability to moderate and ultimately prevent an outbreak from spreading. The question remains as to whether or not there exists some combination of radii that achieves a performance closer to NMIZ than MIZ while still allowing trade between farms in the control zone. To answer this, we introduce various sets of optimised zone radii for both national and regional simulations, as in the previous section. We then offer evidence for the appropriation of Bayesian optimisation in this problem and quantify to what extent it may be used to exploit regional features for economic benefit (without sacrificing stability in controlling the outbreak).

As outlined in “Bluetongue simulations” section, we distinguish between a *training* and *testing* phase. In the former, we use a single year of movement and temperature data (the *train conditions*), together with the Bayesian optimisation framework, to derive radii that minimise the expected number of infected farms. Note that the choice of which year to use for movement and temperature data need not be the same. In the testing phase, we take these radii and run many simulations to get an estimate of the performance metrics under a different set of *test conditions*. The results for both training and testing conditions will be quoted for each experiment. Together, these help assess whether the solution generalises well to unseen events and is an indication of its practicability.

#### National

We begin with outbreaks originating in any region of England and Wales; as in “Benchmark policies” section. Bayesian optimisation was applied to find combinations of radii that minimise the expected number of infected farms, $\mathbb {E}\left[ J^\text {NI}\right]$. Movement and temperature datasets from 2006 and 2013 were used during training to derive the following control radii:

4 $$\begin{aligned}&\text {2006}: \quad \qquad r_\text {CZ}= 6.81\,\text {km},&\quad r_\text {PZ}= 27.31\,\text {km}, \end{aligned}$$

5 $$\begin{aligned}&\text {2013}: \quad \qquad r_\text {CZ}= 2.31\,\text {km},&\quad r_\text {PZ}= 16.48\,\text {km}. \end{aligned}$$

These policies were then evaluated on the opposing datasets in order to assess how well they generalise to unseen events; i.e. the 2006 policy on the 2013 datasets, and the 2013 policy on the 2006 datasets. The performance is summarised in Table 2 in terms of the usual metrics. We find that they compare well with NMIZ in both cases despite having greatly relaxed constraints on movement and much smaller zone sizes. They also appear to exhibit a great reduction in the pseudo-economic cost across both datasets, though there are some discrepancies. In particular, we find that—due to having larger zone sizes—the 2006-derived policy is more costly. This is a manifestation of the running term in Eq. (2), which is incurred at each time step and scales with the size of each zone.

#### Regional

**Table 5:** Central tendency for the number of infected farms and maximum spread distance for 7 regions in the UK using $J^\text {NI}$-optimised control radii for a 2-zone containment policy.

Region	$r_\text {CZ}$ (km)	$r_\text {PZ}$ (km)	Infected farms (–)	Spread distance (km)
Cheshire	4.34	18.47	$1174_{-188}^{+125}$	$45.51_{-12.24}^{+24.36}$
East Sussex	5.01	85.83	$1113_{-70}^{+58}$	$31.32_{-0.77}^{+1.40}$
Hampshire	6.72	56.59	$556_{-67}^{+43}$	$28.06_{-0.73}^{+2.05}$
Norfolk	5.82	16.74	$248_{-50}^{+47}$	$24.16_{-0.91}^{+1.67}$
Somerset	3.44	11.02	$2480_{-279}^{+148}$	$45.69_{-7.06}^{+12.80}$
Dyfed	3.34	11.48	$1195_{-137}^{+161}$	$38.03_{-7.27}^{+11.68}$
Cumbria	7.93	74.79	$307_{-91}^{+109}$	$27.51_{-4.79}^{+21.39}$

These radii were derived from movement and temperature data from 2013; quoted below. Each test simulation was then also run using movement and temperature data from 2013, with the first infection being introduced on day 121 of 365. Estimates are given by the median of 250 samples and are quoted with the 95% confidence interval derived from bootstrapping.

**Table 6:** Central tendency for the number of infected farms and maximum spread distance for 7 regions in the UK using $J^\text {NI}$-optimised control radii for a 2-zone containment policy.

Region	Infected farms (–)	Spread distance (km)
Cheshire	$1376_{-91}^{+100}$	$36.56_{-4.87}^{+11.35}$
East Sussex	$1572_{-56}^{+66}$	$36.10_{-0.78}^{+1.31}$
Hampshire	$758_{-75}^{+80}$	$33.10_{-0.91}^{+1.58}$
Norfolk	$361_{-51}^{+53}$	$27.85_{-0.81}^{+1.80}$
Somerset	$2914_{-151}^{+205}$	$42.99_{-3.26}^{+10.77}$
Dyfed	$1375_{-175}^{+255}$	$31.58_{-1.72}^{+6.92}$
Cumbria	$328_{-124}^{+145}$	$29.03_{-4.32}^{+13.07}$

These radii were derived from movement and temperature data from 2013; quoted in Table 5. Each test simulation was then run using movement data from 2013 and temperature data from 2014, with the first infection being introduced on day 121 of 365. Estimates are given by the median of 250 samples and are quoted with the 95% confidence interval derived from bootstrapping.

**Figure 2:**
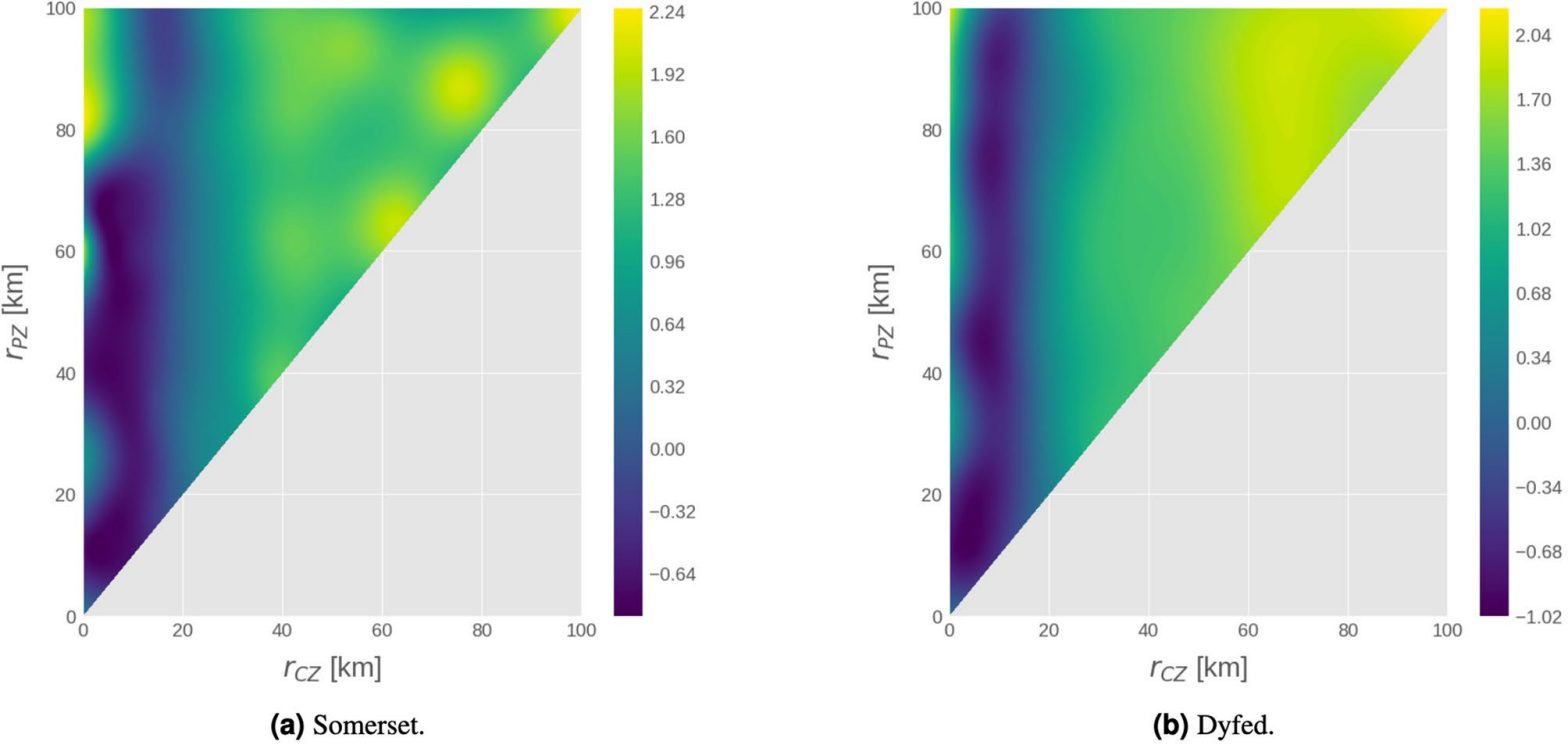
Surrogate regression models generated by the Bayesian optimisation routine after 100 iterations of sampling the simulator. The *x*/*y* axes refer to the radii of the control and protection zones, respectively. The z-axis gives the (standardised) expected number of infected farms, $\mathbb {E}\left[ J^\text {NI}\right]$, for the associated combination of $r_\text {CZ}$ and $r_\text {PZ}$ according to the Gaussian process regression model. The scale of these values is given on the right of each diagram; note that the *values are standardised* due to the dataset transformation specified in “Our model” section. Simulations were performed using movement and temperature data from 2013.

Bayesian optimisation was applied in the same way as in the national case to simulations in each of the 7 regions in Table 1. The radii derived from 2013 movement and temperature data are quoted in Table 5. We found that the performance of the policy is most sensitive to the radius of the control zone. For example, Fig. 2 illustrates two cases where a continuum of optima exist for values around $r_\text {CZ}\approx 5$ km. While the exact value of $r_\text {CZ}$ varies from region to region—typically falling within the range $[4,\, 8]$ kilometres—the pattern appeared throughout; see the supplementary material for more details. This would suggest that the size of the innermost zone (i.e. $\sim 5$ km) is by far the most important factor in reducing the number of infected farms when movement is allowed. This may be attributed to boundary effects near the edges of the range of vector dispersal: when the zone is too small, the midges are able to breach the zones; when the zone is large, the area at risk is also much larger (scaling polynomially with the radius).

**Figure 3:**
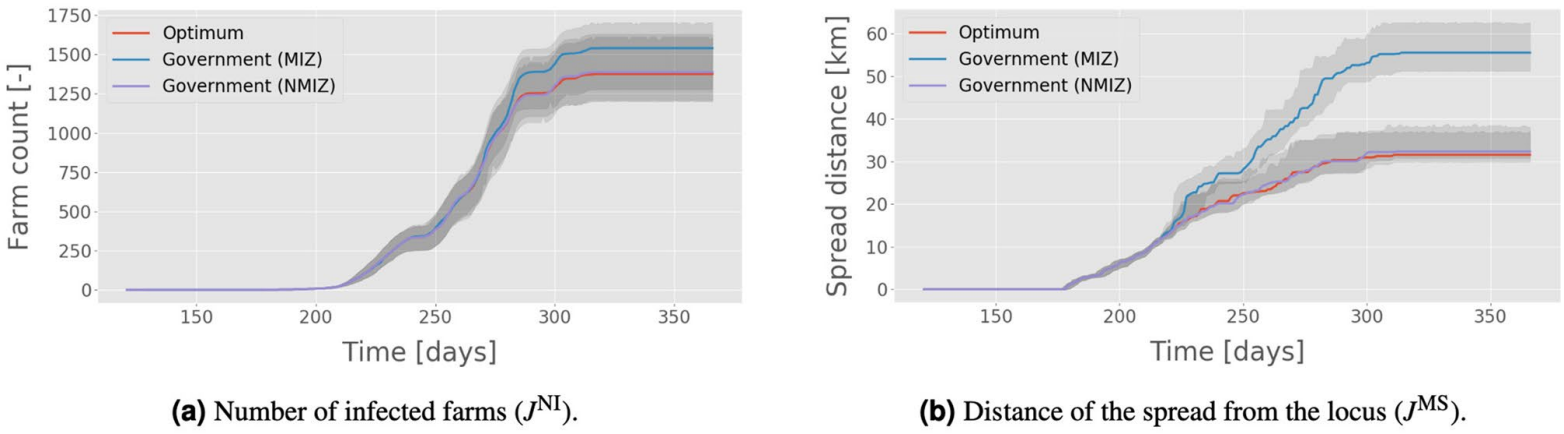
Time series evolution of the median of two metrics over 250 Monte Carlo samples. Each simulation was evaluated in Dyfed with movement data from 2013 and temperature data from 2014; the policy was trained on both movement and temperature data from 2013. Three policies are illustrated: government with (MIZ) and without (NMIZ) movement in the innermost zone, and the $J^\text {NI}$-optimised policy. Uncertainties are given by the 95% confidence interval of the median from bootstrapping.

As in the previous sections, the performances of each OPT solution are given for both the training and testing conditions: movement data from 2013 with temperature data from both 2013 and 2014; see Tables 5 and 6, respectively. In both cases, the $J^\text {NI}$-optimised solutions achieve the same, if not better, levels of containment than the government NMIZ policy in terms of $J^\text {NI}$ and $J^\text {MS}$. This is particularly well illustrated by the time series distributions in Fig. 3 shown here for the Dyfed region; see Section 2 of the supplementary material for the full distributions generated by the simulator in each region. These results further support the argument that neither absolute movement restrictions on farms located in the control zone, nor having three zones as opposed to just two, are needed to effectively mitigate an outbreak.

Finally, we evaluated the performance of the OPT policies on data entirely unseen during the optimisation process. This is a much closer representation of a real-world scenario in which we may only condition our decision making strategy on *information about past events*. To do this, we applied the optimisation procedure to movement and temperature data from 2006 to derive a new set of regional zone radii. These were then evaluated as before on data from 2013, the results of which are summarised in Table 7 in terms of the number of infected farms and maximum spread distance. The former was found to vary negligibly between the two sets of radii (2006 and 2013), and while the spread distance does vary more, it is still insignificant on average. This suggests that the progression of the outbreak is not highly sensitive to the precise values for the radii. Instead it is sufficient that they fall in the broad areas of optimality, as shown in Fig. 2; this is explored further in “Sensitivity analysis” section. Indeed, it is highly probable that the little variation actually observed is just a consequence of the probabilistic nature of the problem, especially given the size of the confidence intervals.

**Table 7:** Central tendency for the number of infected farms and maximum spread distance for 7 regions in the UK.

Region	$r_\text {CZ}$ (km)	$r_\text {PZ}$ (km)	Infected farms (–)	Spread distance (km)
Cheshire	4.59	18.04	$1174_{-182}^{+115}$	$45.51_{-12.24}^{+24.36}$
East Sussex	4.31	11.95	$1106_{-57}^{+67}$	$31.21_{-0.75}^{+1.61}$
Hampshire	9.84	51.12	$557_{-68}^{+39}$	$28.96_{-1.11}^{+1.23}$
Norfolk	4.03	23.84	$248_{-48}^{+50}$	$24.32_{-0.98}^{+2.02}$
Somerset	5.59	19.17	$2488_{-245}^{+172}$	$45.17_{-8.02}^{+13.07}$
Dyfed	2.80	13.11	$1193_{-135}^{+160}$	$37.41_{-5.36}^{+12.60}$
Cumbria	5.90	14.94	$307_{-91}^{+109}$	$26.85_{-3.82}^{+13.74}$

Each simulation was run using movement and temperature data from 2013 with radii derived from data collected in 2006. The first infection was introduced on day 121 of 365 in each case. Estimates are given by the median of 250 samples and are quoted with the 95% confidence interval derived from bootstrapping.

### Economic impact

The economic cost of running a containment policy, $J^\text {EC}$ (see Eq. (3) in “Performance metrics” section), was modelled by a linear combination of immediate and running penalties due to holding animals under a movement ban; the full specification is given “Performance metrics” section. The weights of the model were chosen by hand:

$$w_\Delta = 100$$
A large immediate cost is generated by each newly infected farm.
$$w_{\{\text {PZ},\,\text {SZ}\}} = 1$$
A cost per unit time is applied to farms located in either the protection or surveillance zones, where movement is not fully restricted.
$$w_\text {CZ} = 5$$
A higher cost per unit time is applied to farms located in the control zone, where movement is strictly prohibited. When movement is allowed, as with MIZ and OPT, this value reduces to 1 (as for $w_{\{\text {PZ},\,\text {SZ}\}}$).

These values were chosen to approximate the scale of the economic impact and provide a relative measure by which to compare the three approaches. A full economic model and precise estimation of the real-world impact was beyond the scope of this paper.

**Table 8:** Summary statistics for economic cost of the NMIZ, MIZ and OPT strategies for each of the 7 regions, evaluated on movement and temperature data from 2013.

Region	OPT	MIZ	NMIZ
Cheshire	$0.92_{-0.07}^{+0.12}$	$13.74_{-0.92}^{+0.55}$	$12.96_{-0.64}^{+0.71}$
East Sussex	$1.33_{-0.06}^{+0.02}$	$4.71_{-0.17}^{+0.27}$	$4.42_{-0.21}^{+0.15}$
Hampshire	$1.19_{-0.11}^{+0.07}$	$6.94_{-0.36}^{+0.36}$	$6.77_{-0.28}^{+0.45}$
Norfolk	$0.18_{-0.03}^{+0.01}$	$3.00_{-0.18}^{+0.10}$	$2.98_{-0.17}^{+0.12}$
Somerset	$0.94_{-0.05}^{+0.08}$	$13.85_{-0.44}^{+0.55}$	$12.65_{-0.27}^{+0.62}$
Dyfed	$0.65_{-0.05}^{+0.04}$	$12.03_{-0.87}^{+0.48}$	$10.72_{-0.48}^{+0.39}$
Cumbria	$1.94_{-0.35}^{+0.22}$	$6.11_{-0.77}^{+0.52}$	$5.63_{-0.57}^{+0.78}$

The OPT radii were derived from the same 2013 data. Estimates are given by the median of 250 samples and are quoted with the 95% confidence interval derived from bootstrapping, in units $10^6$.

**Figure 4:**
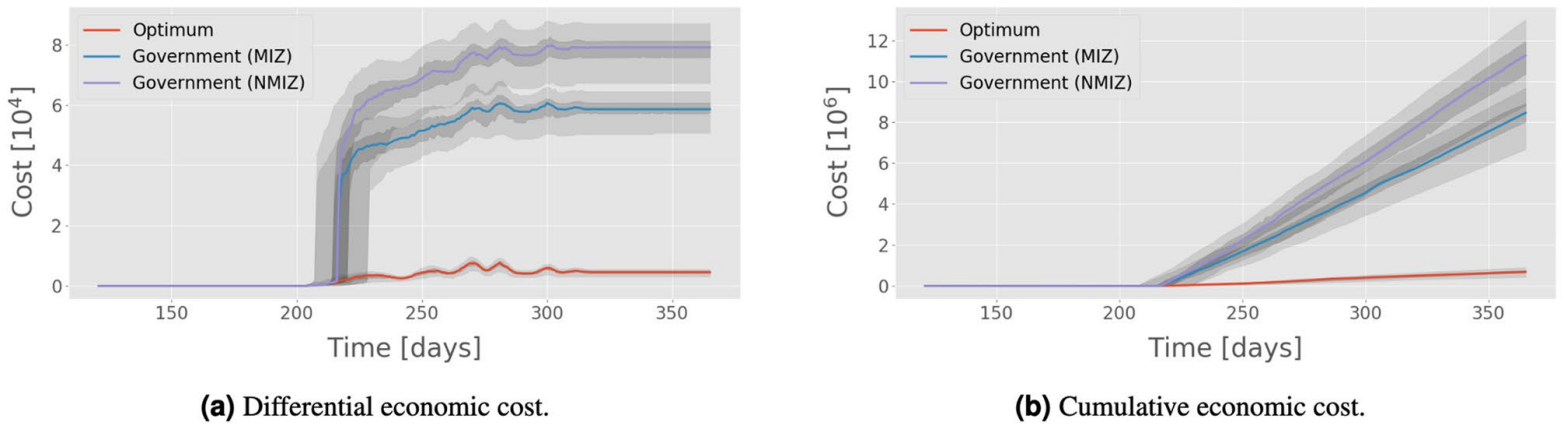
Economic cost of three different policies in Dyfed using movement data from 2013 and temperature data from 2014: government with movement (MIZ) and no movement (NMIZ) in the control zone, and an OPT policy trained on only 2013 data. The cost weights were set to $w_0 = 100,\, w_1 = 5,\, w_{\{2,3\}} = 1$, and uncertainties given by the 95% confidence interval of the median from bootstrapping (darker shaded regions) and the interquartile range (lighter shaded regions).

The results (Table 8) suggest that while both strategies have similar performance, the optimisation-based zones offer a marked reduction in the cost to farms. In many cases this discrepancy is as much as an order of magnitude. To illustrate why this is the case, we examined the evolution of cumulative and differential economic cost (DEC) for the three policies over time; see Eq. (2) for a mathematical definition of DEC. Figure 4 shows one such example, generated through sampling of the Dyfed region. As one would expect, there is an initial period following the start of the spread in which the cost for all policies per unit time is low. Then, after approximately 80 days, there is a change of “phase” leading to a rapid increase in the differential cost of the MIZ and NMIZ policies. It is at this point that the first infections transition from a hidden state to being observable. This phenomenon is also present in the evolution of the number of infected farms and distance of the spread, as shown in Fig. 3. The change in cost per time step pertains to the application of the three zones, which, for the benchmark variants, cover large areas of land which grow at a polynomial rate. As the infection spreads across England and Wales, so the cost per time step increases until an equilibrium is reached.

Figures 3 and 4 provide insight into the functional form of the spread dynamics of BT on a macro scale. When the total number of infections at time *t*, $N_t$, is large compared with $w_\Delta$, the value of the DEC will generally be dominated by the running term in Eq. (2) (i.e. the cost per time step of enforcing zone restrictions). Ignoring the initial linear phase and subsequent discontinuity at day $\sim 220$, we see that the DEC is a sublinear and (approximately) monotonically increasing function of time for all three policies. This implies that the cumulative cost grows at a superlinear rate. Indeed a polynomial of the form $ax^d$, with $d > 1$, is found to produce a reliable fit for the data. Taking the above example, and instead looking at the cumulative sum of the total number of quarantined farms at time *t* (which is functionally equivalent to letting $w_\Delta = 0$ and $w_{\{\text {CZ},\,\text {PZ},\,\text {SZ}\}} = 1$), we find the following: (1) the constant *a* is almost two orders of magnitude smaller for the OPT policy than the benchmark variants; (2) the exponent, *d*, is on the order $\sim 1.07$ for NMIZ and MIZ; (3) the exponent is considerably greater at $d \sim 1.3$ for the OPT policy. These results together suggest that while the performance of the NMIZ/MIZ policies is better in the limiting case of time tending to infinity, the scale of the term *a* dominates the gradient within the finite time horizon considered; and indeed any realistic time horizon. In other words, we can be confident that the cost of the OPT policy will never exceed that of the two alternatives in any realistic scenario. This strongly supports the case for 2-zone, optimised policies given the real constraints on the lifespan of an outbreak of BT.

### Sensitivity analysis

#### Sensitivity to radii perturbations

The optimisation-derived policies, due to their smaller zone sizes, greatly reduce the cost of containment while still constraining outbreaks as effectively as NMIZ. It is thus important to justify these by investigating the impact of perturbations in the enforced radii. We aim to establish whether our policies are susceptible to large changes in performance for small changes in the values of $r_\text {CZ}$ and $r_\text {PZ}$. This requires an understanding of the local gradient of the chosen performance metric $J(r_\text {CZ},\, r_\text {PZ})$. We perform this analysis using a local grid search around the OPT radii in order to form a 3-point numerical estimation of the gradient; perturbations of 0.1 km and 1.0 km were used for the control and protection zone radii, respectively. The following estimates for the local gradients of $J^\text {NI}$ and $J^\text {MS}$ in the Dyfed region (chosen for having the smallest radii) are given by:

6 $$\begin{aligned} \frac{\partial J^\text {NI}}{\partial r_\text {CZ}} \approx -6.553,&\qquad \frac{\partial J^\text {NI}}{\partial r_\text {PZ}} \approx 0.155, \end{aligned}$$

7 $$\begin{aligned} \frac{\partial J^\text {MS}}{\partial r_\text {CZ}} \approx -1.209,&\qquad \frac{\partial J^\text {MS}}{\partial r_\text {PZ}} \approx -0.004. \end{aligned}$$

Comparing these values to the results quoted in Tables 5 and 6 provides a sense of scale and suggests the following: (1) neither $J^\text {NI}$ nor $J^\text {MS}$ are sensitive to changes in $r_\text {PZ}$; (2) the rate of change of $J^\text {NI}$ as a function of $r_\text {CZ}$ of $\sim 0.5\%$ variation per kilometre is negligible compared to the typical number of infected farms for any given sample; (3) the sensitivity of $J^\text {MS}$ to changes in $r_\text {CZ}$ is significant, with a variation on the order of $3\%$ of the typical values per kilometre change in the radius.

These results are intuitive, confirming that performance is more sensitive to $r_\text {CZ}$ than $r_\text {PZ}$, and indicating that small zone sizes are more susceptible to local perturbations. While our proposed zones do appear to be robust overall, this observation will be an important consideration for practitioners. For example, the sensitivity $\partial J^\text {MS}/ \partial r_\text {CZ}$ suggests that restrictions must be upheld strictly by all farms within 1 km of $r_\text {CZ}$. The existence of “defectors” may have a significant impact on the long-term progression of the outbreak. In future work, one could run simulations with a population of these “defector” agents who behave stochastically near zone boundaries. This would certainly form a more robust estimate (and could be integrated into the optimisation process itself), but it is unclear how to construct an appropriate model of farm behaviour; it is unreasonable to assume we have data on this effect.

#### Sensitivity to introduction date

The economic impact of BT increases as a function of the breadth of the spread; i.e. the number of infected farms and the size of the movement-restricted zones. As such, one would expect that the central tendency of the cost increases as a function of the length of the period of communicability. To characterise this effect, the OPT radii derived in “Regional” section were re-evaluated on simulations where the date of introduction of BT was moved 30 days later to May 31st; again, movement and temperature data from 2013 were used. In doing so, we reduce the total amount of time available for the outbreak to propagate before winter. The results are summarised in Table 9, which gives the average economic cost over the 7 regions for the OPT and NMIZ policies. In contrast to our expectations, it was found that the average cost *increased* when the date of introduction was set to May 31st. This shows that a longer period of infection does not necessarily lead to a larger outbreak.

**Table 9:** Economic cost averaged over 7 regions for BT introduction dates of May 1st and May 31st. 250 simulations were run using movement and temperature data from 2013 with the OPT radii given in Table 5.

Date of introduction	OPT $\left[ 10^6 \right]$	NMIZ $\left[ 10^6 \right]$	$\frac{\texttt {OPT}}{\texttt {NMIZ}}$ ratio
May 1st	1.02	8.02	0.16
May 31st	1.14	8.62	0.16

Interestingly, we find that while the cost may vary between the two starting dates, the *ratio* between the two policies remains approximately the same (to 2 significant figures). This suggests that the effectiveness of the $J^\text {NI}$-optimised policies relative to NMIZ is not lost when the introduction date is moved, *even if the optimisation process is not repeated for the new start date*. This robustness does not mean that performance couldn’t necessarily be improved by taking the start date into account explicitly in the optimisation process. One could derive many instances of the control policy and apply each based on the observed/presumed starting conditions of an outbreak. However, in comparison with the impact of radii perturbations and regional features (as we shall see next), the potential gains observed in our experiments do not appear to be statistically significant and further simulations would be needed to make such a claim.

#### Sensitivity to spatial features

**Figure 5:**
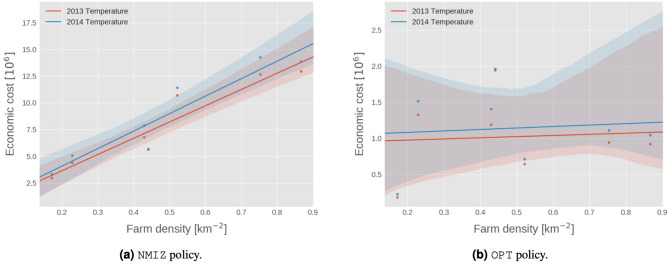
Regression plot between the density of farms within 100 km of the centre of each region and the estimated economic cost for the government (NMIZ) and derived policies. Evaluation was performed on movement data from 2013, and temperature data from both 2013 and 2014. The one OPT policy was trained on 2013 data only. Error bands are derived from bootstrapping and form the 95% confidence interval on the regression.

Past work has indicated that the propagation of BT infections does not persist unless there are between-farm vector transmissions which are highly dependent on the spatial distribution of farms. Indeed, the relationship between the total pseudo-economic cost—which itself is directly related to the size of the spread—and the density of farms within 100 km of the region centre for NMIZ is evidence of this; see Fig. 5. As shown in Fig. 7 in the supplementary material, we do observe a relationship between the OPT radii themselves and farm densities. Yet, while the OPT control radii (Table 5) do themselves vary between regions, we do not find statistically significant evidence of correlation between the economic cost and either the area of each region, or the farm density; see Fig. 5. While it is hard to draw strong conclusions from Fig. 5, it may suggest that there are intrinsic properties of each region which dominate the variation in the spread of the virus and supersede simple, univariate measures of structure. These may be attributed to species composition, temperature patterns or spatial clustering of farms. By enforcing regional policies, one may account for these effects—either implicitly during optimisation or explicitly—and minimise the variation in performance based on where the outbreak began. It may also encourage further work looking at the microstructure of the problem, such as farm-to-farm interactions and policies which leverage this information explicitly.

## Discussion

The original paper upon which this work is based—that of Turner et al.^18^—showed that an outbreak of bluetongue can be effectively mitigated through the use of local animal movement restrictions. Rather than a unilateral ban between all farms, it was shown that a single 20 km control zone, or 100 km protection zone, would perform as effectively without affecting every farm. Our motivation was two-fold: first, to show that much more compact, and thus more economically viable, policies may be derived directly from data via optimisation; and second, to highlight the importance of contextual information in tailoring the response to an outbreak. In so doing, we showed that one can reduce cost to farms by specialising policies to the region of origin with no reduction in performance.

To begin, we established a benchmark performance estimate from evaluations of two benchmark policies. We then proposed a framework for optimising control radii using Bayesian optimisation for a 2-zone version of the MIZ policy. The derived solutions were shown to compete with the highly restrictive NMIZ approach, both in terms of the number of infected farms as well as the lateral spread distance of the outbreak. This was shown to be the case on both the national (i.e. outbreaks starting in random regions) and regional basis. The robustness of these results was also confirmed by testing the sensitivity of the zone radii to perturbations and variation in the conditions under which the outbreak occurred.

The first conclusion to draw from these results is that one cannot hope to do significantly better than the NMIZ (Defra) control policy in terms of containment. The size of the three zones and highly restrictive constraints on movement in the control zone effectively subdue an outbreak. Instead, we show that one can achieve *equivalent* performance with greatly reduced restrictions on animal movement. These results generalise well and, while the radii can vary significantly, evidence suggests that models based on past data provide a good basis upon which to derive policies. This extends to the regional case as well: using policies trained on simulations of outbreaks in a specific region yields significant cost reductions. This is particularly relevant in regions with a relatively high farm density such as Cheshire, Somerset and Dyfed.

An open question remains, however, about the source of variation in the OPT control radii. Correlation was only observed between the radii and farm densities, not with animal densities nor spatial coordinates. This does not appear to tell the whole story; after all, correlation is not causation. Instead, it might suggest that there are other factors driving the spread of bluetongue—and thus the optimisation process—that are unique to each region. This is well illustrated by comparing the surrogate model surfaces generated by the optimisation process; see the optimisation surfaces in the supplementary material, paying particular attention to Cumbria. It is plausible that the spatial clustering of farms, the structure of trade networks and/or local weather patterns will have an impact on the resulting policy. We argue that this supports the need for *contextual epidemiological responses*—a finding reflected in past work^25^. Such policies could, as we show, restrict animal movements effectively while minimising the economic cost on farms. Our characterisation of the cost dynamics (as defined in Eq. 3) show that this effect may be as much as an order of magnitude; indeed, every plausible combination of weights that could be used in the model result in this same conclusion. It is important to note, however, where these regional artefacts may play a negative role. In general, the optimal solution will vary due to the following natural and artificial artefacts:

1. Farm distributions and movement networks vary significantly from region to region which may lead to very different optimal responses. These characteristics, however, do tend to be consistent from year to year. As illustrated during testing, the policies learnt from past data appear to generalise well to future conditions.
2. The movements of animals within a given network tends to be sensitive to temperature. An important open question to address is how this will worsen over the coming decades in response to climate change. Incorporating seasonal forecasts into the model might improve robustness to extreme events, but this relies on accurate predictions.
3. The use of finite datasets on animal movements may lead to a bias in the optimisation process. This can be mitigated through the use of cross-validation and/or testing on hidden datasets as we have shown in this work.

To test robustness, we performed a sensitivity analysis of three susceptible components of our approach to disturbance: perturbations in the radii, and sensitivity to the introduction date and spatial features of each region. In the first case, only a small impact was observed in the performance following perturbations about the solution. While not negligible, the size of the variation in radii would suggest that our policies could be realistically implemented nationwide. After all, even the effort of maintaining a control zone—the most sensitive zone to perturbations—of radius $\sim 3.4$ km pales in comparison to that of the 20 km prescribed by NMIZ. In the second case we saw that the optimisation method is robust to different introduction dates and even found that the justification for our approach will only grow as temperatures in the UK grow under the influence of climate change; a risk shown to be all too real in recent work^7^. Finally, by showing that the economic performance of the OPT radii is insensitive to a region’s farm density, we strengthen the argument that more efficient methods may be designed within the current policy framework.

It is also important to mention that other control methods do exist besides movement restrictions in the form presented here. These approaches are often complementary and may be implemented simultaneously: take vaccination, for example. Indeed, any effective response to an outbreak of Bluetongue should be multi-faceted in order to be robust. However, it is worth noting that, historically, this has not been the case. In 2008, Defra encouraged the use of vaccination, but left farmers to bear the brunt of the cost, leading to variable coverage. Furthermore, it is often impossible to tell whether an animal has been infected or vaccinated. This has a significant impact on decision-making. The focus of this work was to establish whether less economically devastating approaches can be taken that are equally as effective. We leave it to future work to quantify the impact of combining both movement restrictions and vaccination together, and extending our optimisation model to this setting.

The main advantage of our approach is that it exploits the availability of data about the movement of animals between farms and temperatures in the UK. With the growing prevalence of high fidelity data, many opportunities for the development of empirical, data-driven approaches to epidemiological control problems such as this are becoming more feasible. Moreover, these methods are typically very general. The specific framework for optimisation introduced in this work may be applied to simulators of other diseases that are susceptible to spread via animal movements with little-to-no modification. Bayesian optimisation is a particularly effective technique for tackling problems of this type. Due to its high sample efficiency it is capable of quickly identifying performant solutions, reducing the need for extensive sampling of the simulator (which is costly) and minimising the amount of data needed before reasonable inferences can be made (again reducing cost). This is especially true when reliable knowledge about the problem is available beforehand as it may be encoded in the optimiser in the form of a prior. The internal surrogate (Gaussian process) models used by the optimiser may also be exploited in their own right as emulators for the various measurable quantities generated by the simulator^33^. This would enable institutions to distribute software to farms that provide information about their exposure to risk during an outbreak. These decentralised models would circumvent issues around data privacy since the end users do not need access to the movement or temperature data. Gaussian processes are also much faster to query than sampling a full stochastic model, are much easier to implement and require less computational resources.

In this paper we have argued that the large fixed zone schemes currently implemented to contain outbreaks of bluetongue are far from efficient. The costs on farms, even under favourable assumptions, are enormous and may very well have disastrous consequences in their own right. Using optimisation techniques, we have shown that one does not need to sacrifice performance or practicability to make a significant reduction in cost. We argue that the added complexity of choosing radii based on the initial infection region incurs only a negligible burden on those tasked with applying this in the real world; though our technique is also shown to generalise well. In the long term, the impacts of climate change on our environment will require us to look at how one can apply these techniques to more and more epidemiological challenges that pose a threat to humans and animals in the UK.

## Optimisation model

### Gaussian process regression

Regression is the process of finding a mapping from some input $\mathbf {x}$ to a continuous response variable *y*. For example, in this paper the input $\mathbf {x}$ is a vector of control radii and the output *y* is the expected number of affected farms during a single outbreak of bluetongue. The standard linear model for such problems defines the relationship between $\mathbf {x}$ and *y* as the product of the input variables with a real vector of weights $\mathbf {w}$,

8 $$\begin{aligned} f(\mathbf {x}) = \mathbf {x}^\top \mathbf {w}, \quad y = f(\mathbf {x}) + \varepsilon . \end{aligned}$$

Typically, one assumes that the observed values differ from the function output, $f(\mathbf {x})$, only by an additive noise term $\varepsilon$, and that the noise is independently and identically distributed according to a Gaussian distribution; i.e. $\varepsilon \sim \mathcal {N}(0, \sigma _n^2)$. While many efficient methods exist for solving linear regression problems of this form^30^—such as maximum likelihood estimation (of which ordinary least squares is a special case) and Bayesian inference—the assumption of linearity means that these models only capture a very limited number of problems.

One approach to modelling more complex relationships is through a *basis function expansion* of (8). In this case, the input $\mathbf {x}$ is replaced by a possibly non-linear basis function $\mathbf {\phi }(\mathbf {x})$, amounting to a projection of the input onto some higher dimensional space. We call the output of $\mathbf {\phi }(\cdot )$ the *feature vector*. One such example is polynomial regression which uses a set of basis functions of the form $\phi _j(x) = x^j$. For many real problems, however, it can be unclear how to construct or even compute an appropriate basis without introducing some form of bias and issues of overfitting. In cases where there is some notion of similarity between inputs, we may instead take the approach of defining a *covariance function*, or *kernel*, $k(\mathbf {x}, \mathbf {x}')$. Many algorithms have been developed that exploit this structure explicitly via the so-called *kernel trick*, such as Support-Vector Machines^30^ and Gaussian process (GP) regression (GPR)^34^, the latter being the technique used in this work.

Gaussian process regression is a tool for making predictions about unseen data without specifying an explicit model for $f(\mathbf {x})$. Instead, we use a Gaussian process as a Bayesian prior for expressing the beliefs about the underlying function we are trying to capture. Formally, a GP is a collection of random variables, any finite number of which have a joint Gaussian distribution^34^; GPs may be thought of as the extension of the multivariate Gaussian distribution to infinite dimensionality. This representation is expressed as $f(\mathbf {x}) \sim GP(m(\mathbf {x}), k(\mathbf {x}, \mathbf {x}'))$, where $m(\mathbf {x})$ is the mean function of the GP and $k(\mathbf {x}, \mathbf {x}')$ is the kernel function mentioned previously. The idea behind GPR is then simple: given some set of training samples, $(\mathbf {X}, \mathbf {y})$, one may reason about the functions producing a probable fit of the data. Rather than infer weights of some assumed model, the goal is to infer a posterior distribution over the space of functions that could plausibly fit the data. This is done by first constructing a joint distribution over the training outputs $\mathbf {y}$ and the test outputs $\mathbf {y}^\star$ according to the prior. Conditioning this distribution on $\mathbf {X}$ and $\mathbf {X}^\star$ then yields the posterior.

Intuitively, the kernel defined in the GP prior provides a measurement of *similarity* between pairs of inputs. It is then assumed that the target values are likely to be close if the value of $k(\mathbf {x}, \mathbf {x}')$ is large for the corresponding input pair (i.e. highly similar inputs). In other words, the training observations closest to some chosen test location provide the most information about the prediction at that point. It is quite clear then that the specification of the kernel function is tantamount to the nature of the model as it implies a distribution over the possible functions we may fit. In most applications the squared-exponential or Matérn kernels^34^ are sufficient and as such are by far the most commonly used in practice.

### Bayesian optimisation

Bayesian optimisation^35,36^ (BO) is a technique for efficiently finding the maxima or minima of functions—known as *objective functions*—which may be very expensive or difficult to evaluate, and for which we do not have access to gradients. One such example is when the function may only be evaluated through sampling of some external simulator. This kind of Monte-Carlo estimation can require a lot of computing power and time. In many cases this can render conventional approaches to optimisation as infeasible. To solve this problem, Bayesian optimisation introduces the notion of a surrogate model, which is a differentiable approximation to the real objective function. The surrogate model is typically given by a GP (see “Gaussian process regression” section) due to its flexibility and convenient analytical properties. A template of the BO process is outlined in Algorithm 1.
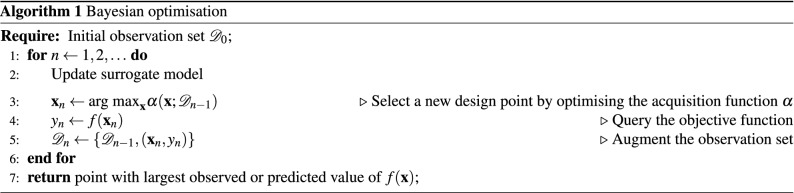


Key to the success of Bayesian optimisation is the definition of the acquisition function, $\alpha (\mathbf {x};\; \mathcal {D})$ (see step 3). This is a function of the surrogate model used to select design points to sample. One of the most commonly used acquisition functions is the *expected improvement* (EI) criterion. This function is literally defined as the expectation of the improvement of a proposed point over the current best observed value. To derive an expression, we first define $y_\star = \min _i y_i$ as current best observation, and then quantify the improvement after sampling a new point $\mathbf {x} \in \mathcal {X}$ as the difference, lower bounded at 0. The EI acquisition function, $a_\text {EI}(\mathbf {x})$ is then given by the following:

9 $$\begin{aligned} a_\text {EI}(\mathbf {x})&= \mathbb {E}\left[ \max {\left( y_\star - f(\mathbf {x}), 0\right) )} \mid \mathbf {x},\, \mathcal {D} \right] , \end{aligned}$$

10 $$\begin{aligned}&= \underbrace{(y_\star - \mu (\mathbf {x})) \Phi (y_\star ; \mu (\mathbf {x}), K(\mathbf {x}, \mathbf {x}))}_\text {Exploration} + \underbrace{K(\mathbf {x}, \mathbf {x})\mathcal {N}(y_\star ; \mu (\mathbf {x}), K(\mathbf {x}, \mathbf {x}))}_\text {Exploitation}. \end{aligned}$$

Equation 10 above shows how EI naturally trades off *exploration* (sampling points with high variance/uncertainty) with *exploitation* (sampling points with good predicted performance). This helps to prevent overfitting in regions with the best predicted performance without exploring areas we know less about given the decay of similarity defined by our kernel. For applications such as epidemiology, and those with high risk, this may be particularly relevant.

Many other choices of acquisition function exist, such as the probability of improvement, upper confidence bound and entropy search to name a few. However, few have as natural an interpretation as expected improvement. In domains where explainability is important, this can be an especially valuable trait. The problem: EI is not suitable when we only have access to noisy observations; that is, where the samples in the dataset are themselves not exact measurements of the function $f(\mathbf {x})$. A common solution to this problem is to use modified variants of EI, such as EI with “plug-in”^37^, augmented EI^38^, or knowledge gradients^39^. These strategies for handling noise range in computational complexity and effectiveness. However, broadly speaking, each has its own set of pitfalls, and choosing between each depends on computational resources and the problem characteristics.

### Our model

We chose to represent the surrogate model in our BO routine with a standard Gaussian process for prediction with noisy observations, as described in most texts^30,34,40^; note that in the following we will be discussing BO in the context of *minimisation*. Without loss of generality, we assume a prior over the mean function, $$m(\mathbf {x})$$, of zero, and standardise the observed target values, $$\mathbf {y}$$, using the mean and standard deviation; this process is re-applied at each iteration of the optimisation loop. That is, we apply a transformation $$\widetilde{y}_i = (y_i - \mu _{\mathbf {y}}) / \sigma _{\mathbf {y}}$$, where $$\mu _{\mathbf {y}}$$ and $$\sigma _{\mathbf {y}}$$ denote the sample mean and standard deviation on $$\mathbf {y}$$, respectively. The noise variance term $$\sigma _n^2$$ was then constrained to lie in the range $$[10^{-9}, 10^6)$$ with initial value taken as a fraction of the empirical variance in the transformed outputs. Finally, a Matern 5/2 kernel was used to define similarity between inputs, as given by the following equations:

11 $$\begin{aligned} k_\nu (\mathbf {x}, \mathbf {x}')&= \frac{1}{\Gamma (\nu ) 2^{\nu - 1}} \left[ \frac{\sqrt{2\nu }}{l}d\right] ^\nu K_\nu \left( \frac{\sqrt{2\nu }}{l}d\right) , \end{aligned}$$

12 $$\begin{aligned} k_{\nu = 5/2}(\mathbf {x}, \mathbf {x}')&= \left( 1 + \frac{\sqrt{5}d}{l} + \frac{5d^2}{3l^2}\right) e^{\frac{-\sqrt{5}d}{l}}, \end{aligned}$$

where (11) is the generalised kernel and (12) is the expanded 5/2 variant that we use. The distance $d = d(\mathbf {x}, \mathbf {x}')$ is defined in Euclidean space; i.e. $d(\mathbf {x}, \mathbf {x}') = \sqrt{\sum _i (\mathbf {x}_i - \mathbf {x}_i')^2}$. Both $\nu$ and *l* are positive parameters, and $K_\nu$ is the modified Bessel function^41^. Note that a GP with this kernel is twice differentiable in its sample paths, yielding an acceptable level of smoothness while being less susceptible to overfitting.

For simplicity, we used EI with “plug-in” as the acquisition function. This scheme uses a different target for improvement compared to standard EI. Specifically, we replace $y_\star$ (the best *observed value*) in (9) with the best posterior *predicted value* amongst all sampled data points: $\min _i \mu (\mathbf {x_i})$. The main limitation of this approach is that we assume zero noise in the future sampled data point. This means that EI with “plug-in” can underperform in regions with high variance. To mitigate this, each observation was taken as the empirical average over 100 samples. We found that this combination had an acceptable balance between computational cost and efficiency of search. In each iteration, the internal optimisation steps were performed using the Broyden–Fletcher–Goldfarb–Shanno algorithm^42^.

#### Software

In this work we made use of a pair of robust software packages that perform Gaussian process regression and Bayesian optimisation, namely **GPy**^43^ and **GPyOpt**^44^.

## Supplementary information

Supplementary information.
