# Supplementary material for: Bayesian optimisation of restriction zones for bluetongue control
Source: Sci Rep. 2020 Sep 15;10:15139. doi: 10.1038/s41598-020-71856-4 (PMC7494917; doi:10.1038/s41598-020-71856-4)
Supplement: Supplementary file 1 — Supplementary information. [file 41598_2020_71856_MOESM1_ESM.pdf]

Bayesian optimisation of restriction zones for bluetongue control

Thomas Spooner[1,*], Anne E Jones[2,4], John Fearnley[1], Rahul Savani[1], Joanne Turner[3], and Matthew Baylis[3]

[1]University of Liverpool, Department of Computer Science
[2]University of Liverpool, Department of Mathematical Sciences
[3]University of Liverpool, Department of Epidemiology and Population Health
[4]IBM Research, The Hartree Centre, STFC Daresbury Laboratory, Sci-Tech Daresbury, Warrington, WA4 4AD
[*]t.spooner@liverpool.ac.uk

ABSTRACT

Supplementary information for the paper “Bayesian optimisation of restriction zones for bluetongue control”. Included are: 1) the model parameters; 2) metric distributions; 3) time series heatmaps; 4) illustrations of the optimisation procedure, and 5) the surrogate models.

1 Bluetongue model specification

Table 1. Parameters, model functions and values used in simulating the spread of bluetongue.[1]

	Description	Values
t_{intro}	Day of the year in which the infection is introduced.	121 / 151
F_{intro}	Farm type of initial infection.	Any (cattle, sheep, or mixed)
T_{cutoff}	Vector activity threshold (equal to ν_2). The temperature below which the vector is inactive.	13.34°C
vecd1	Maximum vector travel distance per day.	15km
r_{CZ}	Radius of control zone.	20km
r_{PZ}	Radius of protection zone.	100km
r_{SZ}	Radius of surveillance zone.	150km
D	Diffusion parameter.	0.531
σ	Feeding preference.	0.5
prev$E$	Proportion of exposed animals on "exposed" farms.	0.01
mixed_ratio	Proportion of cattle to sheep in a mixed herd.	0.33
β_{hv}	Probability of transmission from host to vector given an effective contact.	0.01
β_{vh}	Probability of transmission from vector to host given an effective contact.	0.9
$c_{C,S}$	Host incubation rate (cattle, sheep).	1/7, 1/5
$\nu(T)$	Latent (extrinsic incubation) rate: $\nu(T) = \max\{0, \nu_1(T - \nu_2)\}$, where T is temperature.	
ν_1	Scale factor of temperature-dependent latent rate.	0.019
ν_2	Temperature below which the virus cannot replicate.	13.34
$m(t,T)$	Vector to host ratio: $m(t,T) = \exp\{b_0 + p_1 \sin[\theta(t-\psi_1)] + p_2 \sin[2\theta(t-\psi_2)] + cT\}$, where t and T are time and temperature, respectively. $b_0 = 0$, $p_1 = 10.59$, $p_2 = 3.71$, $c = 0.07$, $\theta = 0.0172$, $\psi_1 = 128.4$, $\psi_2 = 81.7$	
$a(T)$	Vector biting rate $a(T) = \max\left\{0, a_1 T (T - a_3)(a_4 - T)^{1/a_2}\right\}$. $a_1 = 0.0002$, $a_2 = 2.7$, $a_3 = 3.7$, $a_4 = 41.9$	
$\mu(T)$	Vector mortality rate $\mu(T) = \mu_1 e^{\mu_2 T}$. $\mu_1 = 0.009$, $\mu_2 = 0.16$	
$B(T_{\text{exp}})$	Probability of conversion: $B(T_{\text{exp}}) = 1 - e^{-(T_{\text{exp}}/\lambda_C)^{k_C}}$, where T_{exp} is the time since exposure. $\lambda_V = 10$, $k_C = 3$	

$R(d_I)$	Probability of detection: $R(d_I) = 1 - (1 - \lambda_A\, d_I)^H$,<br>where $\lambda_A = \lambda_{D0}$ (or λ_{D1}) for cattle and mixed farms prior (or after) the first detected infection; similarly for sheep with λ_{DS0} and λ_{DS1}. We also define d_I as the prevalence of infection on the farm and H as the herd size.<br>$\lambda_{D0} = 0.001$ $\lambda_{DS0} = 0.001$ $\lambda_{D1} = 0.01$ $\lambda_{DS1} = 0.01$
$d_S(T_{\mathrm{rec}})$	Degree of susceptibility: $d_S(T_{\mathrm{rec}}) = 1 - (1 - (d_{S0} - F/H))\, e^{-(T_{\mathrm{rec}}/\lambda_I)^{k_I}}$,<br>where d_{S0} is the farm's degree of susceptibility prior to its last outbreak, F is the number of infected animals on the farm in last outbreak, and T_{rec} is the time since the farm recovered.<br>$\lambda_I = 912.5$ $k_I = 2$

2 Distributions of outbreak metrics

2.1 Number of infected farms

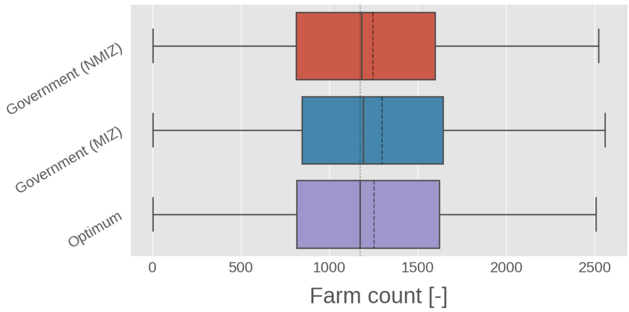

(a) Cheshire

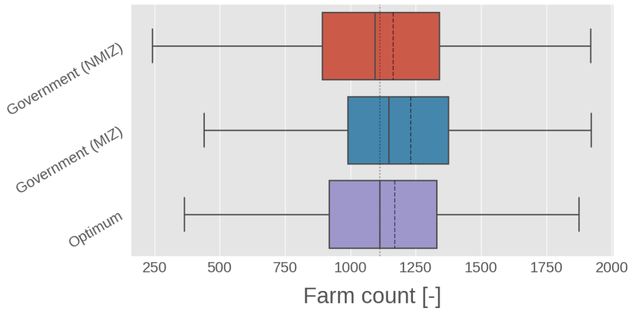

(b) East Sussex

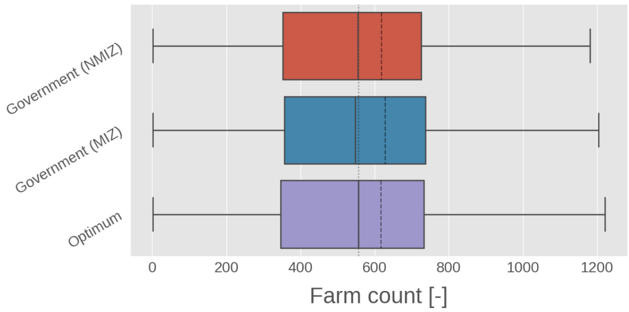

(c) Hampshire

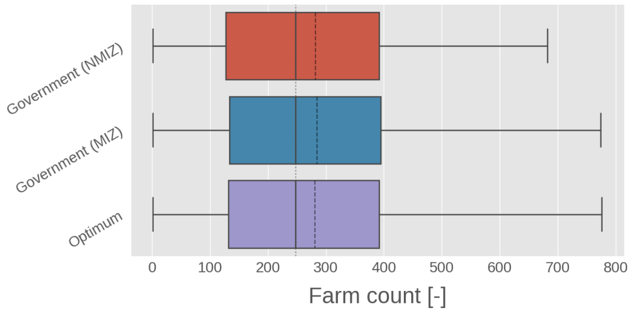

(d) Norfolk

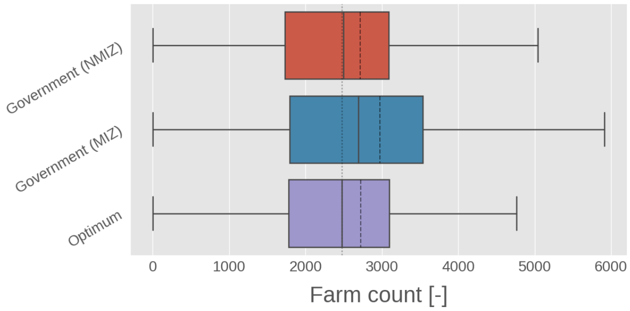

(e) Somerset

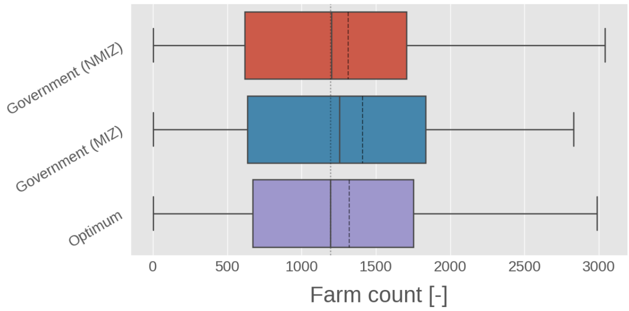

(f) Dyfed

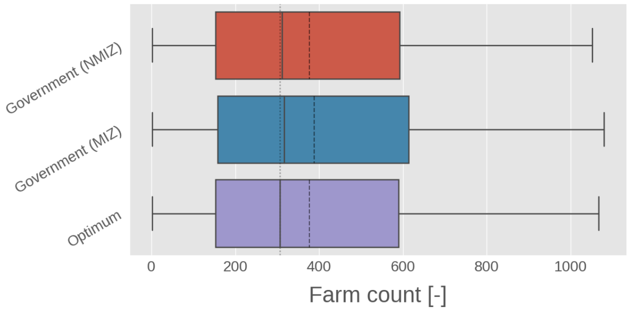

(g) Cumbria

Figure 1. Distribution of the *number of infected farms* across 7 regions for three policies: government with (MIZ) and without (NMIZ) movement in the control zone, and the J^{NI}-optimised policy. Each of the 250 simulations were started with an initial infection on day 121, using movement and temperature data from 2013. The `OPT` policies were trained on 2013 data only. Boxes represent the interquartile range of the distribution with the whiskers covering the full range. The solid and dashed vertical lines correspond to the median and mean, respectively.

2.2 Maximum spread distance

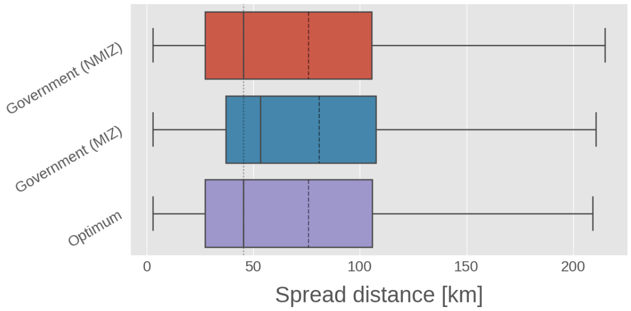

(a) Cheshire

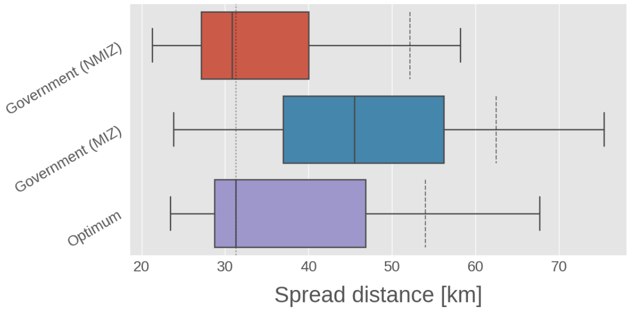

(b) East Sussex

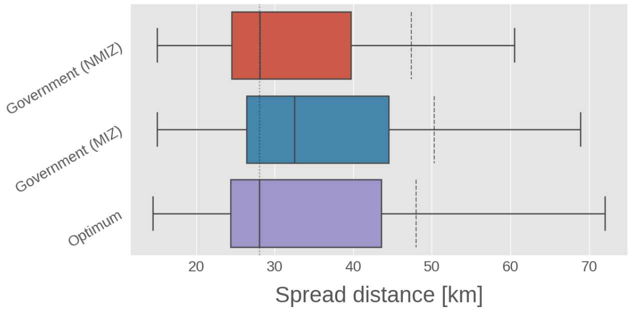

(c) Hampshire

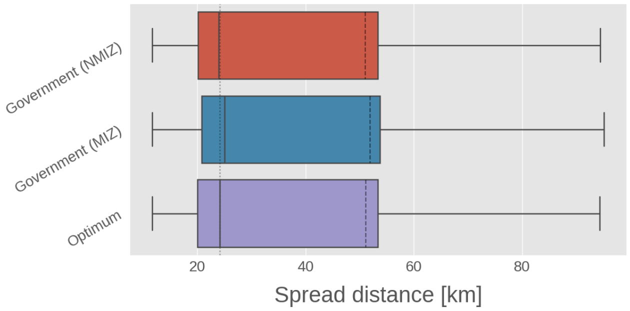

(d) Norfolk

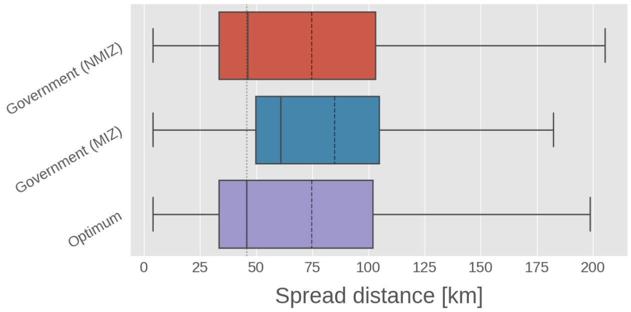

(e) Somerset

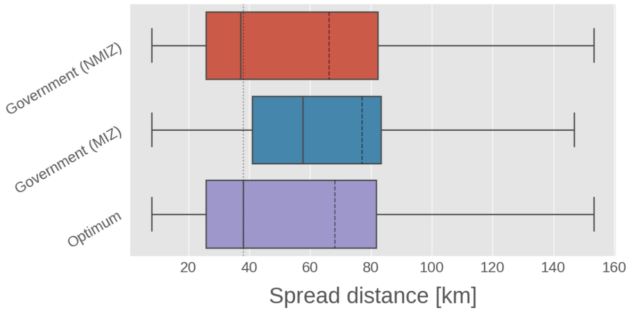

(f) Dyfed

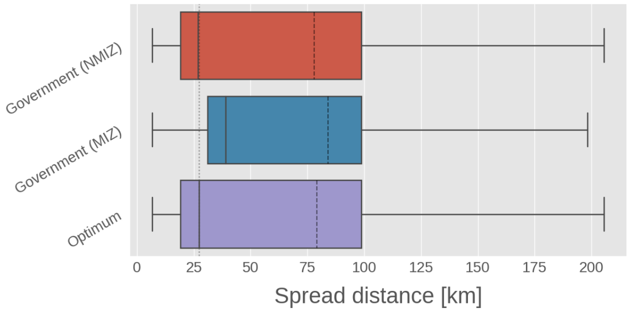

(g) Cumbria

Figure 2. Distribution of the *maximum distance reached by the spread* across 7 regions for three policies: government with (MIZ) and without (NMIZ) movement in the control zone, and the J^{NI}-optimised policy. Each of the 250 simulations were started with an initial infection on day 121, using movement and temperature data from 2013. The `OPT` policies were trained on 2013 data only. Boxes represent the interquartile range of the distribution with the whiskers covering the full range. The solid and dashed vertical lines correspond to the median and mean, respectively.

3 Time series of outbreak metrics

3.1 Number of infected farms

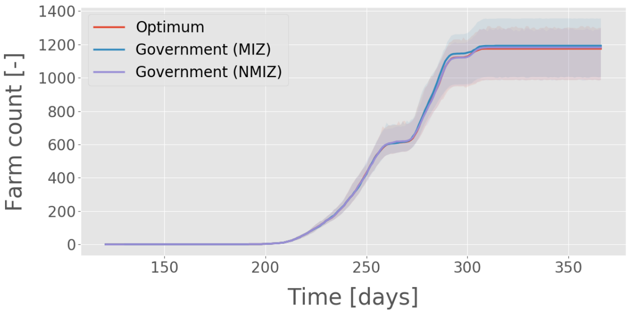

(a) Cheshire

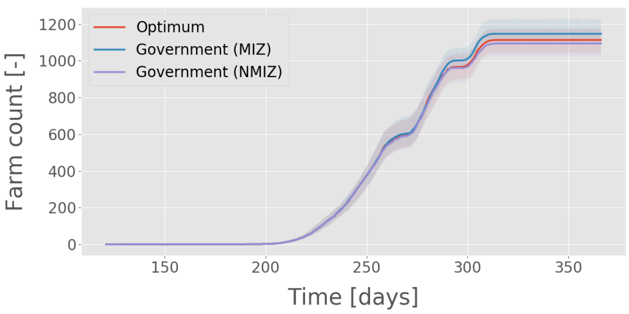

(b) East Sussex

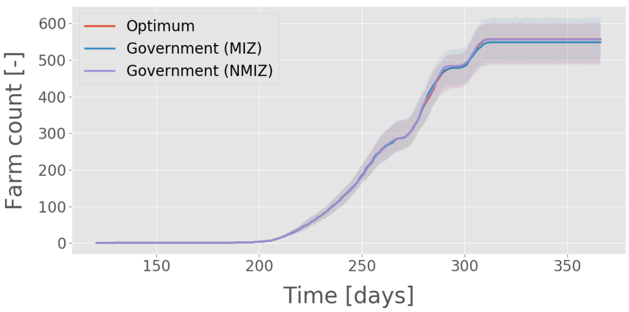

(c) Hampshire

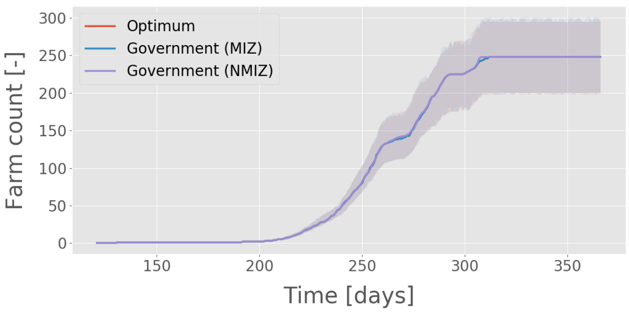

(d) Norfolk

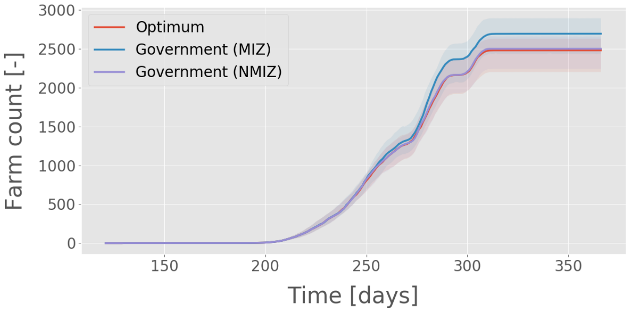

(e) Somerset

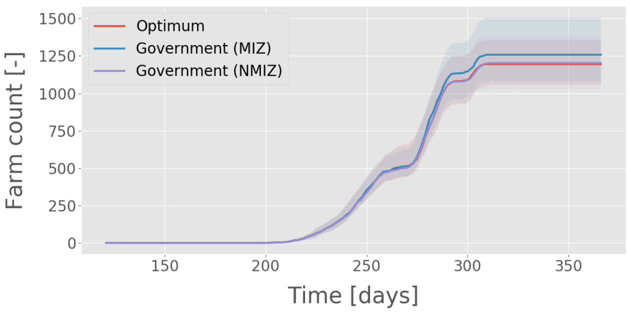

(f) Dyfed

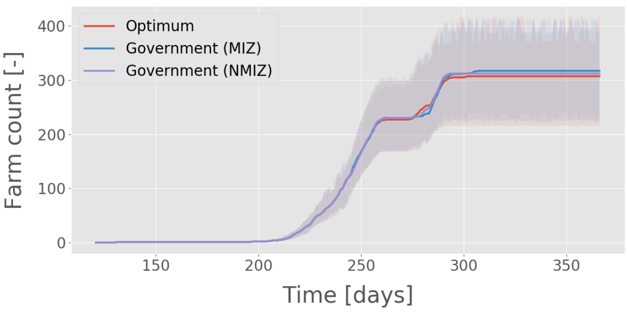

(g) Cumbria

Figure 3. Time series evolution of the *median number of infected farms* across 7 regions for three policies: government with (MIZ) and without (NMIZ) movement in the control zone, and the J^{NI}-optimised policy. Each of the 250 simulations were started with an initial infection on day 121, using movement and temperature data from 2013. The `OPT` policies were trained on 2013 data only. Uncertainties are given by the 95% confidence interval of the median from bootstrapping.

3.2 Maximum spread distance

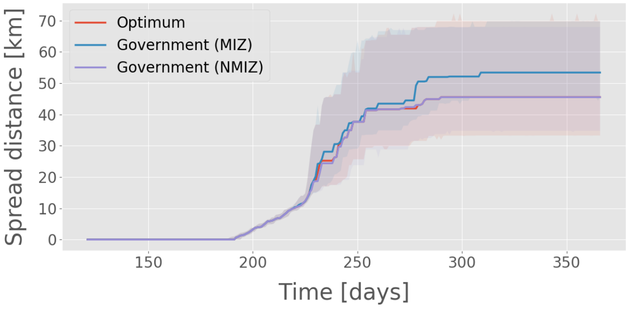

(a) Cheshire

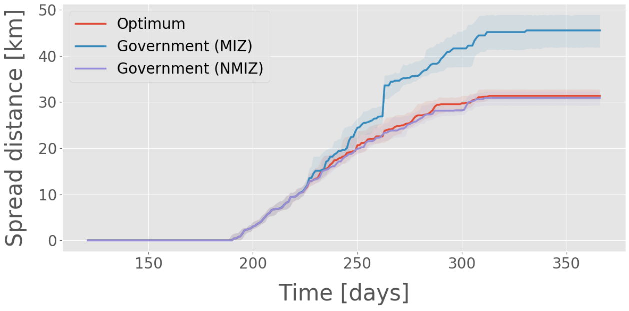

(b) East Sussex

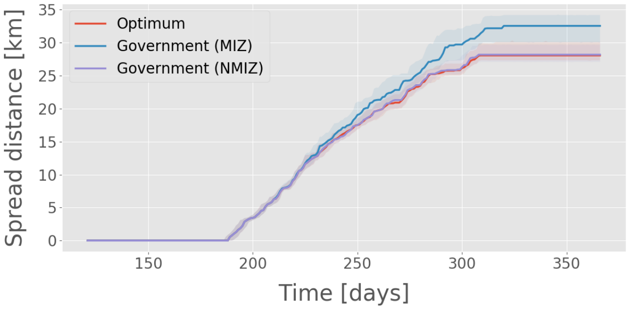

(c) Hampshire

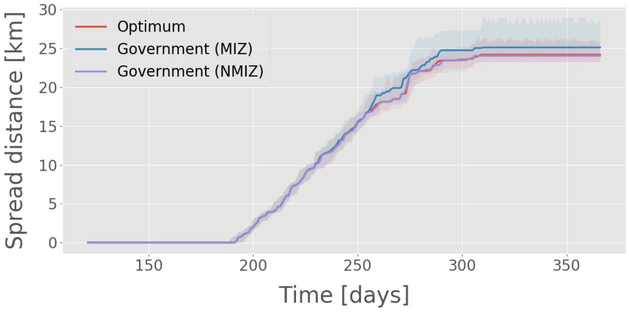

(d) Norfolk

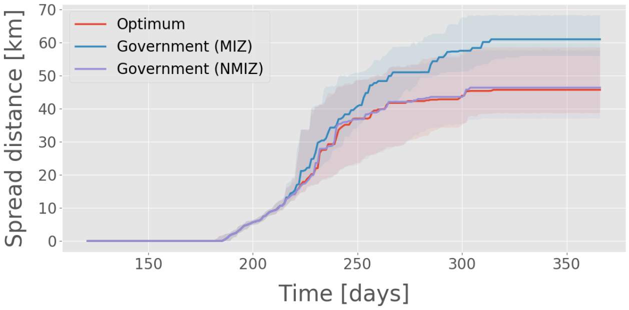

(e) Somerset

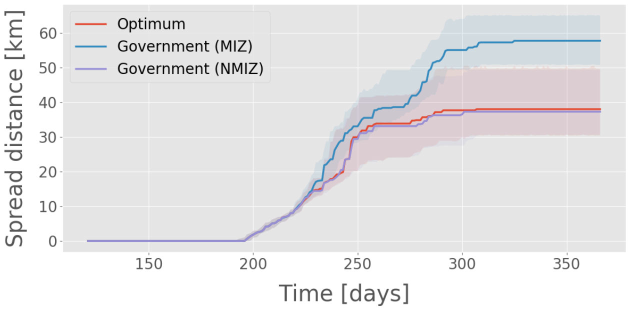

(f) Dyfed

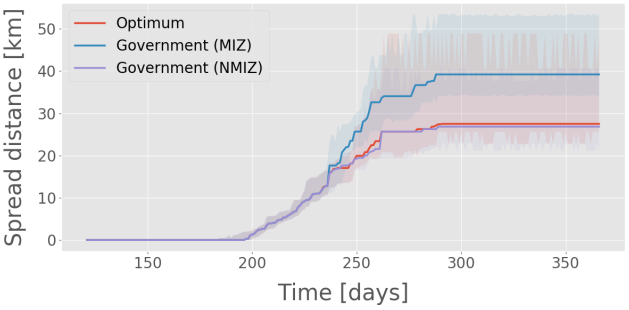

(g) Cumbria

Figure 4. Time series evolution of the *median of the maximum distance reached by the spread* across 7 regions for three policies: government with (MIZ) and without (NMIZ) movement in the control zone, and the J^{NI}-optimised policy. Each of the 250 simulations were started with an initial infection on day 121, using movement and temperature data from 2013. The OPT policies were trained on 2013 data only. Uncertainties are given by the 95% confidence interval of the median from bootstrapping.

3.3 Differential economic cost

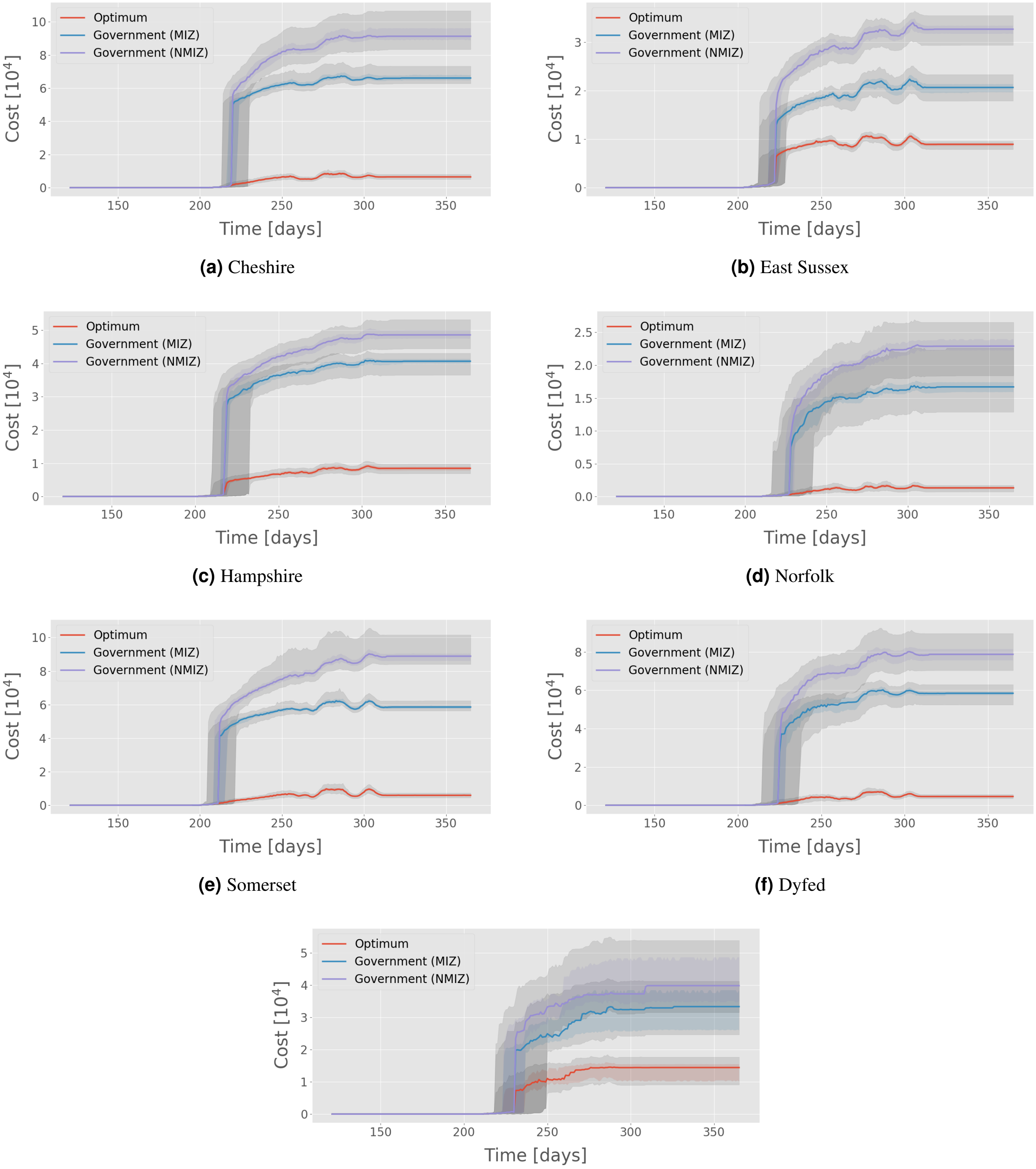

(a) Cheshire

(b) East Sussex

(c) Hampshire

(d) Norfolk

(e) Somerset

(f) Dyfed

(g) Cumbria

Figure 5. Time series evolution of the *median differential economic cost* across 7 regions for three policies: government with (MIZ) and without (NMIZ) movement in the control zone, and the J^{NI}-optimised policy. Each of the 250 simulations were started with an initial infection on day 121, using movement and temperature data from 2013. The `OPT` policies were trained on 2013 data only. The cost weights were set to $w_0 = 100$, $w_1 = 5$, $w_{\{2,3\}} = 1$, and uncertainties given by the 95% confidence interval of the median from bootstrapping (darker shaded regions) and the interquartile range (lighter shaded regions).

3.4 Cumulative economic cost

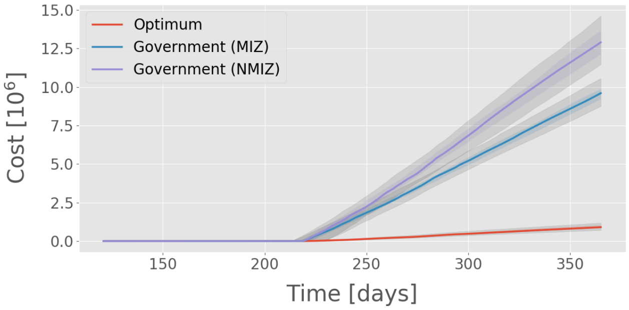

(a) Cheshire

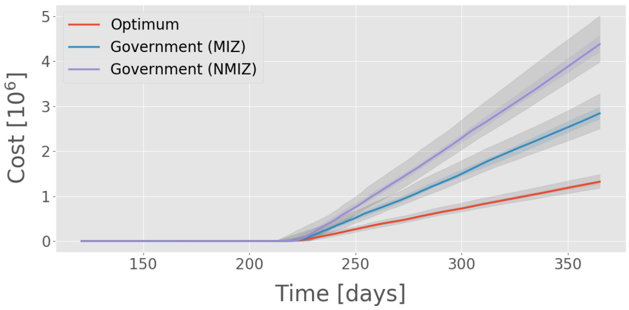

(b) East Sussex

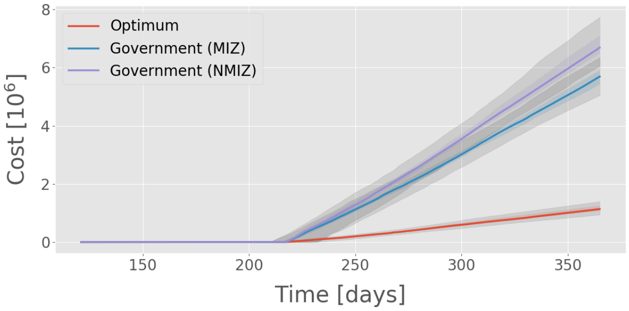

(c) Hampshire

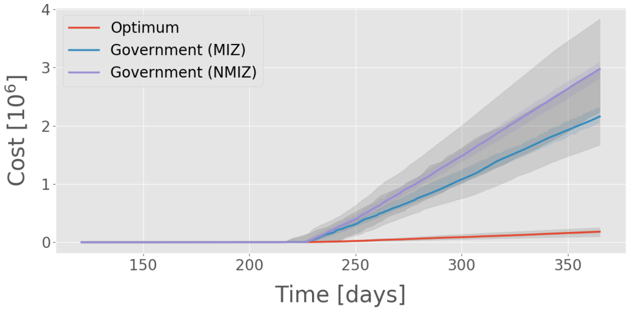

(d) Norfolk

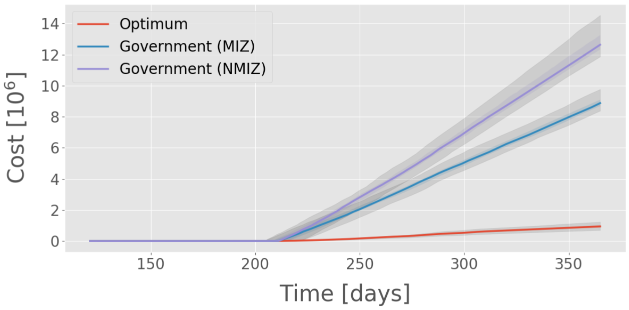

(e) Somerset

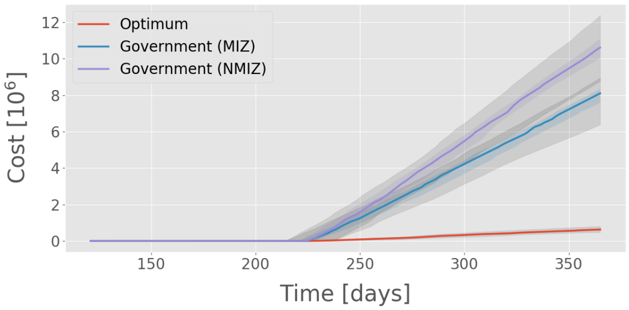

(f) Dyfed

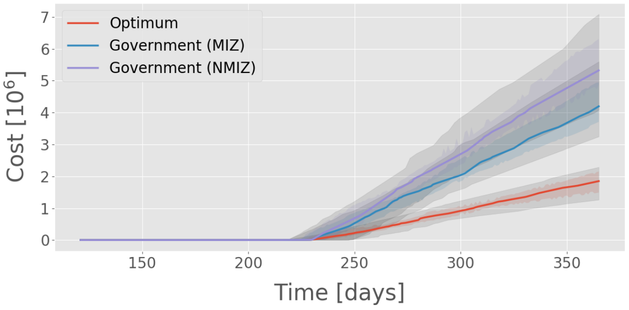

(g) Cumbria

Figure 6. Time series evolution of the *median cumulative economic cost* across 7 regions for three policies: government with (MIZ) and without (NMIZ) movement in the control zone, and the J^{NI}-optimised policy. Each of the 250 simulations were started with an initial infection on day 121, using movement and temperature data from 2013. The `OPT` policies were trained on 2013 data only. The cost weights were set to $w_0 = 100$, $w_1 = 5$, $w_{\{2,3\}} = 1$, and uncertainties given by the 95% confidence interval of the median from bootstrapping (darker shaded regions) and the interquartile range (lighter shaded regions).

4 Radii sensitivity analysis

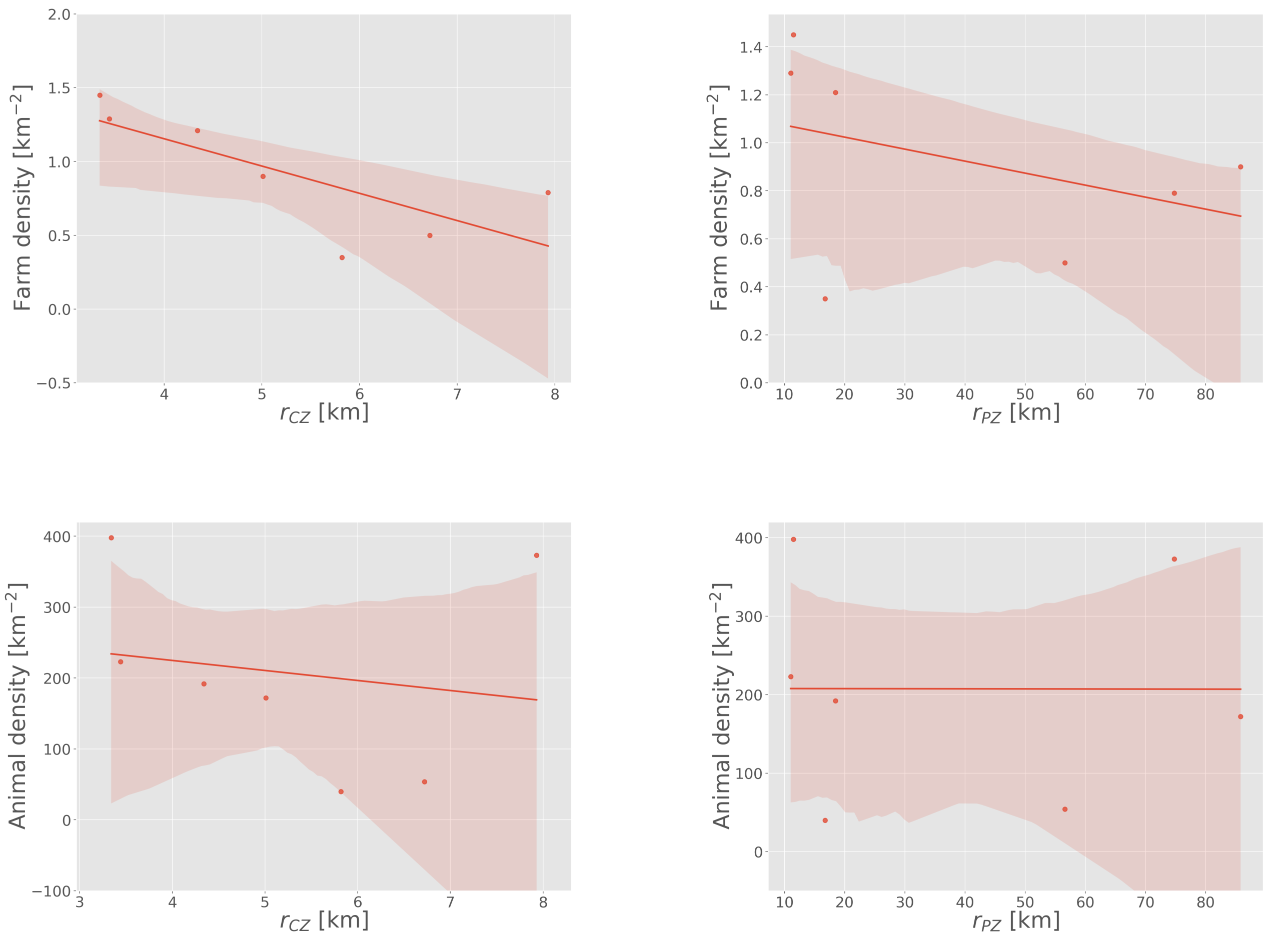

Figure 7. Regression plot between the density of farms/animals within 100km of the centre of each region and the control/protection zone radii derived through optimisation (`OPT`) on 2013 movement and temperature data. Error bands are derived from bootstrapping and form the 95% confidence interval on the regression.

5 Optimisation illustration

Compared to many other methods, Bayesian optimisation is incredibly efficient, requiring few observations to infer a lot about the cost function landscape. Figure 9 shows an example of the rate of convergence of the optimiser in finding the minimum of $J^{\mathrm{NI}}(r_{\mathrm{CZ}}, r_{\mathrm{PZ}})$. Each point corresponds to the mean value of a set of samples from the simulator for a given parameterisation of the control radii. Initially the performance is poor and so large gains ($\sim 5\%$) are made after only 2 observations. The optimiser then makes steady improvements until iteration 40. For problem domains such as epidemiology, where simulators are often very slow to sample, this efficiency is particularly important for practical use.

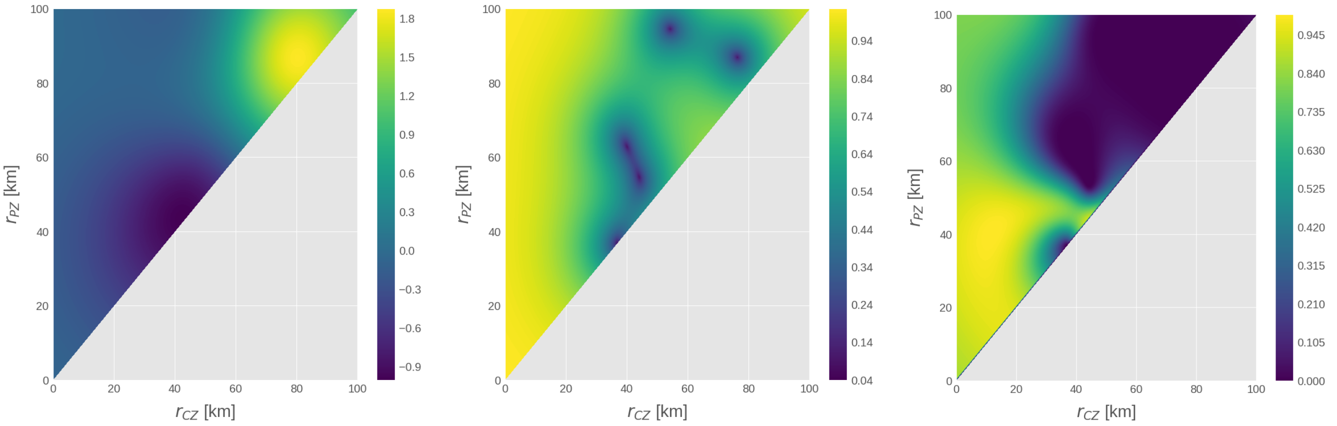

(a) Iteration 5

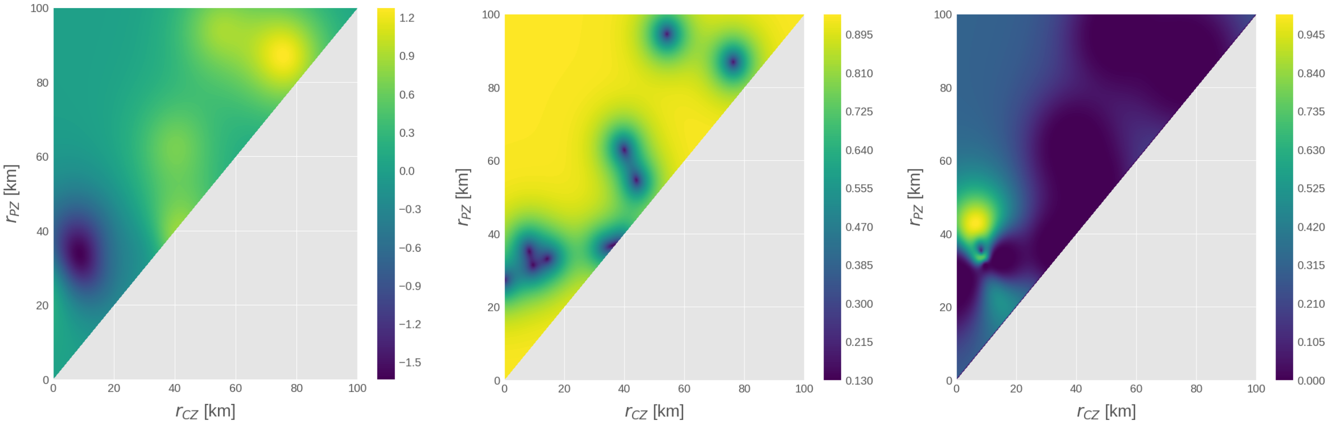

(b) Iteration 10

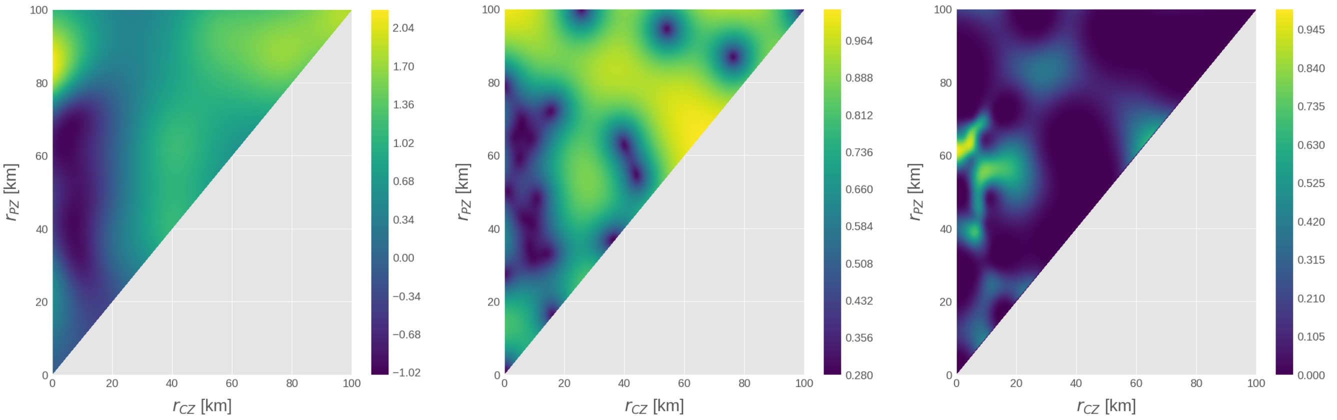

(c) Iteration 25

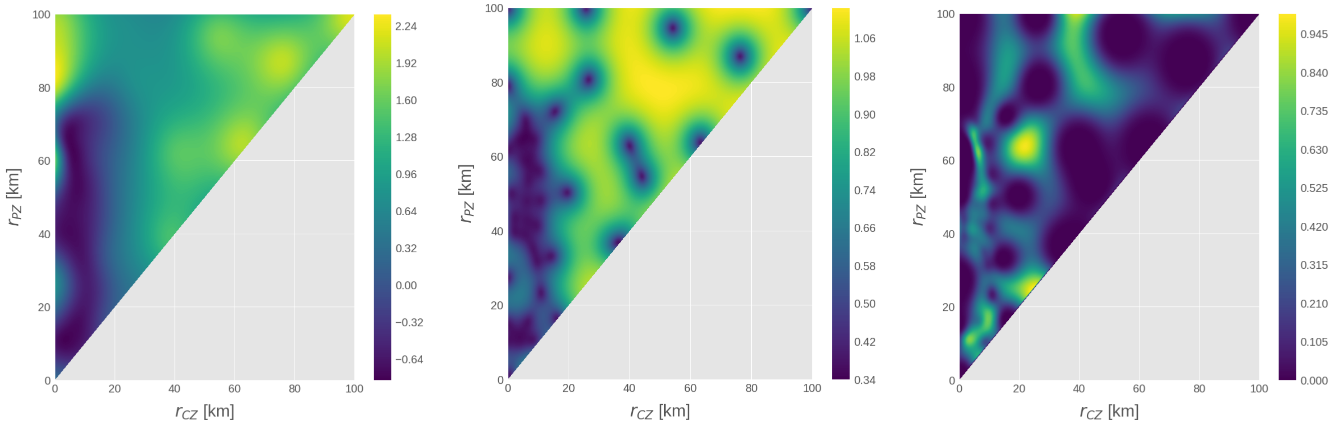

(d) Iteration 50

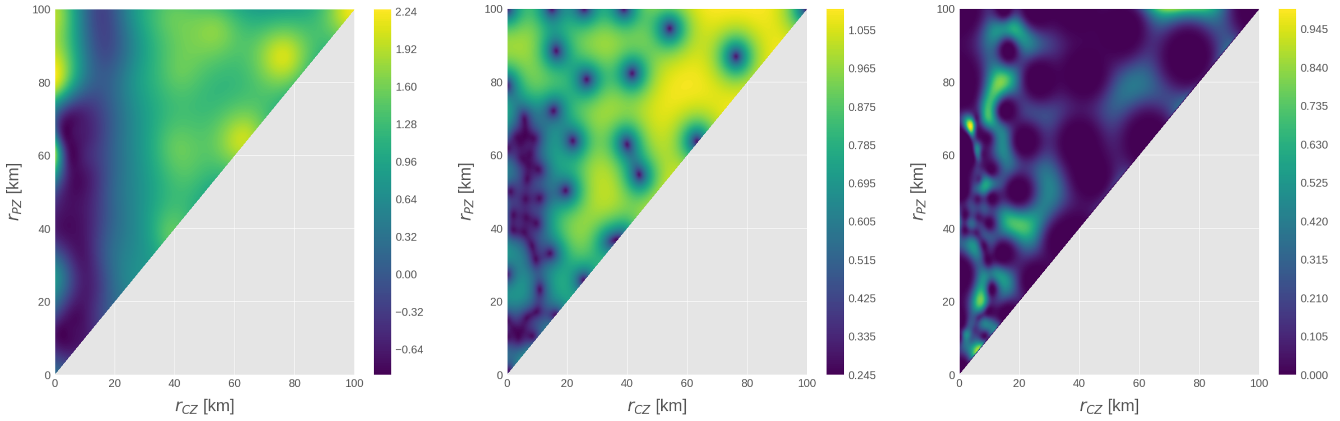

(e) Iteration 100

Figure 8. Bayesian optimisation process applied to simulations of bluetongue originating from Somerset using movement and temperature data from 2013, with infections started on day 121. The three columns depict the mean and standard deviation of the Gaussian process, and the implied expected improvement acquisition function, respectively.

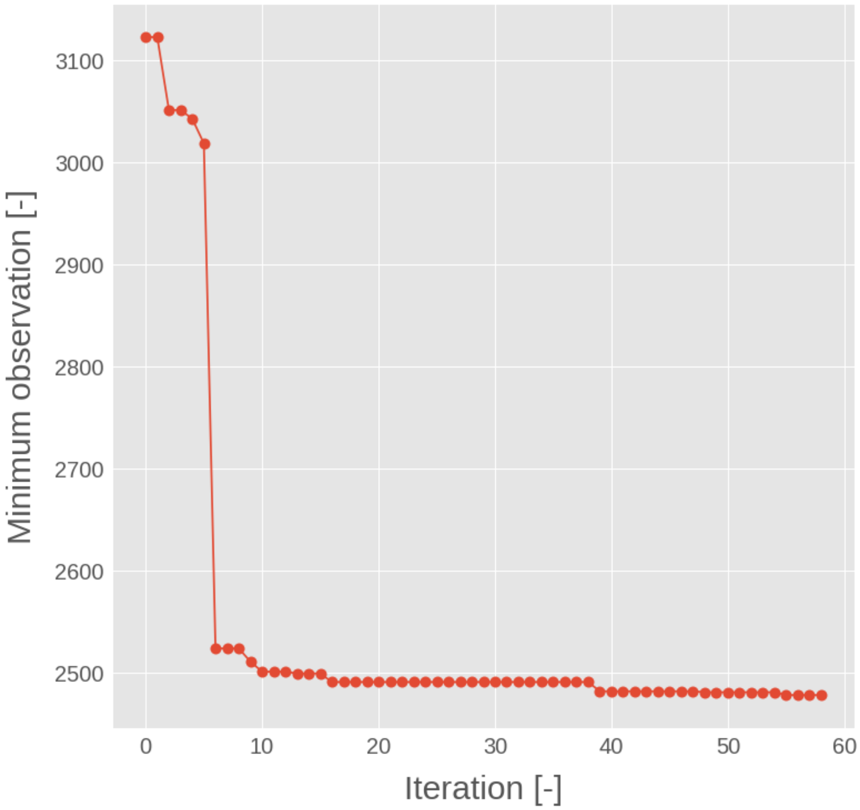

Figure 9. Convergence of Bayesian optimisation on a solution that minimises the expected number of infected farms J^{NI} over 100 iterations of sampling disease outbreaks in Somerset. Each simulation was started with an infection on day 121, using movement and temperature data from 2013.

6 Surrogate models

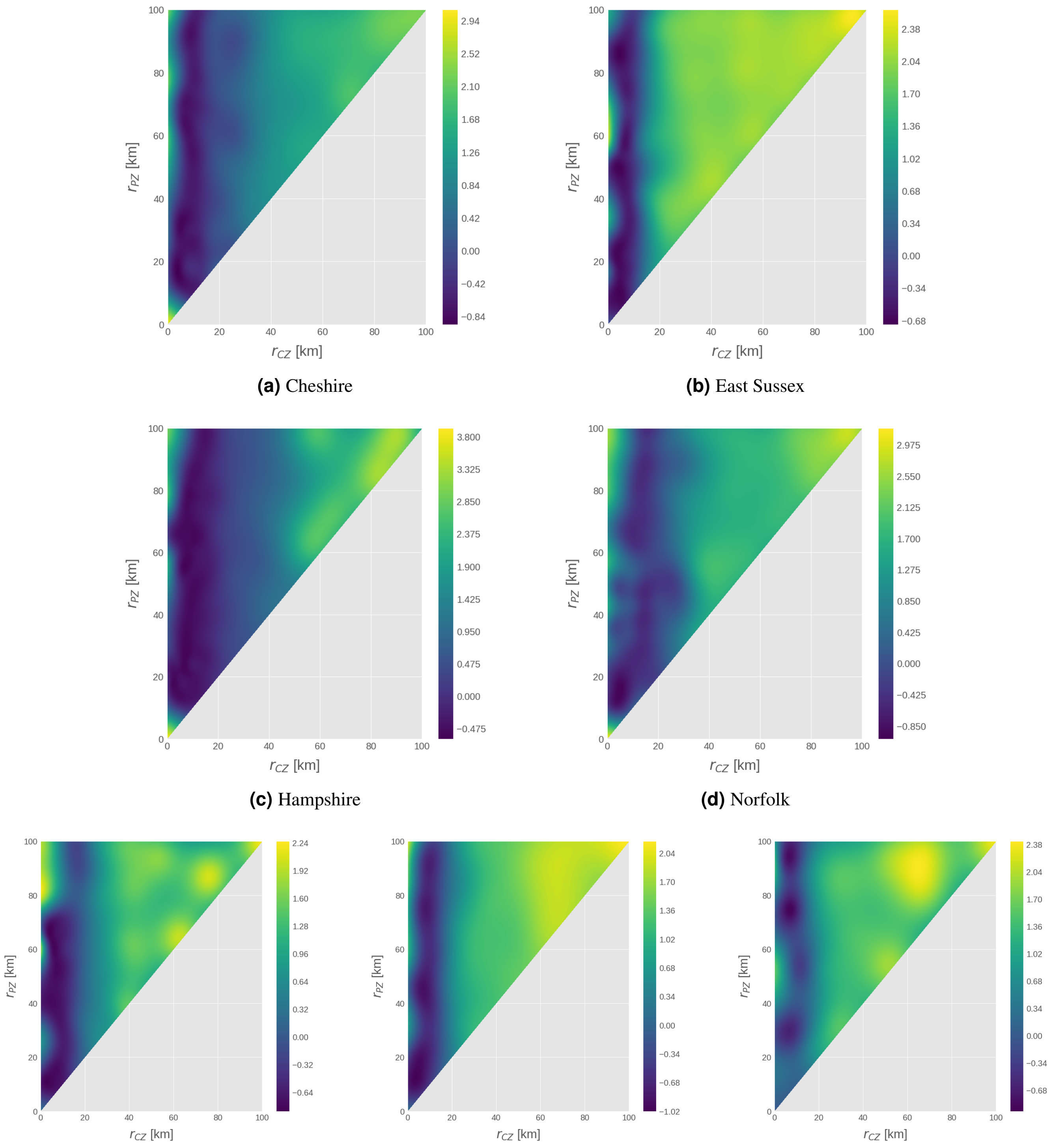

(a) Cheshire **(b)** East Sussex

(c) Hampshire **(d)** Norfolk

(e) Somerset **(f)** Dyfed **(g)** Cumbria

Figure 10. Regression models for the *expected value for the number of infected farms* generated by the Bayesian optimisation routine after 100 iterations of sampling; note that the z-axis is normalised. Each simulation was started with an initial infection on day 121, using movement and temperature data from 2013.

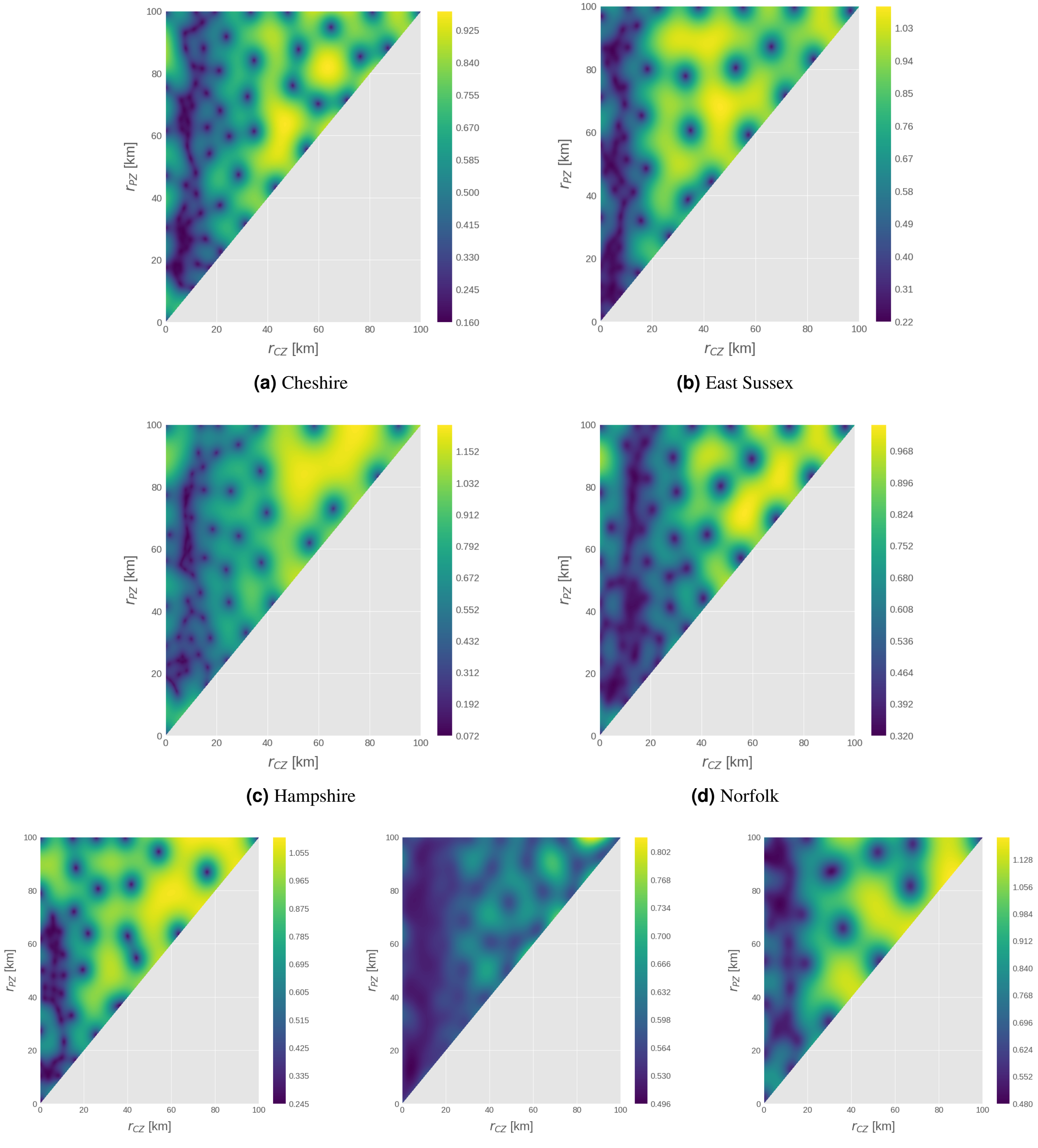

(a) Cheshire **(b)** East Sussex

(c) Hampshire **(d)** Norfolk

(e) Somerset **(f)** Dyfed **(g)** Cumbria

Figure 11. Regression models for the *standard deviation on the expected number of infected farms* generated by the Bayesian optimisation routine after 100 iterations of sampling; note that the z-axis is normalised. Each simulation was started with an initial infection on day 121, using movement and temperature data from 2013.

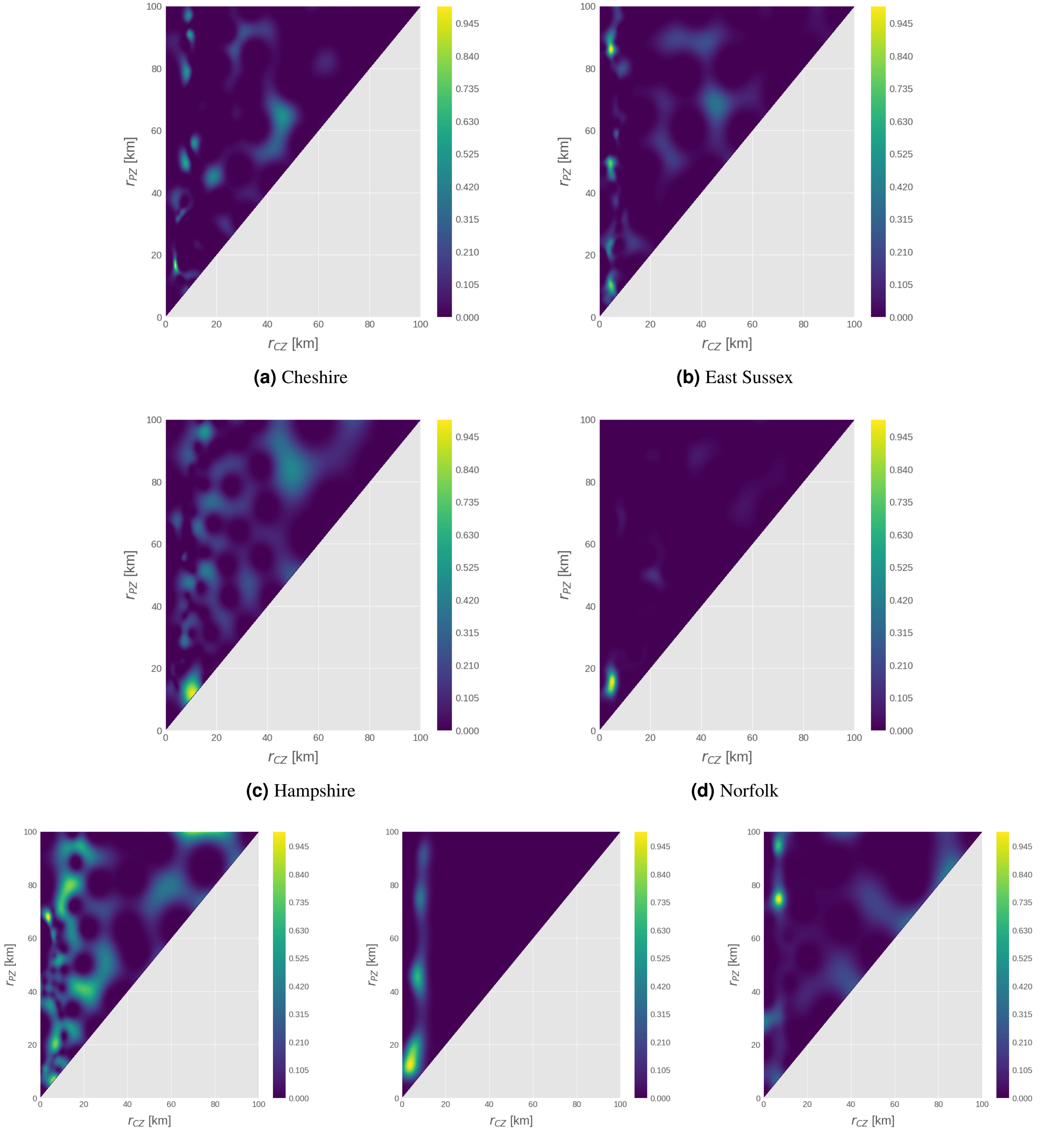

(a) Cheshire **(b)** East Sussex **(c)** Hampshire **(d)** Norfolk **(e)** Somerset **(f)** Dyfed **(g)** Cumbria

Figure 12. Expected improvement acquisition functions derived from the surrogate models generated by the Bayesian optimisation routine after 100 iterations of sampling; note that the z-axis is normalised. Each simulation was started with an initial infection on day 121, using movement and temperature data from 2013.

References

1. Turner, J. *et al.* The effect of temperature, farm density and foot-and-mouth disease restrictions on the 2007 UK bluetongue outbreak. *Sci. Reports* **9**, 112 (2019).
